# Single-cell protein activity analysis reveals a novel subpopulation of chondrocytes and the corresponding key master regulator proteins associated with anti-senescence and OA progression

**DOI:** 10.3389/fimmu.2023.1077003

**Published:** 2023-03-23

**Authors:** Zhao Guang, Zhang Min, Li Jun-Tan, Dou Tian-Xu, Gao Xiang

**Affiliations:** ^1^ Department of Orthopedics, The Fourth Hospital of China Medical University, Shenyang, China; ^2^ Department of Obstetrics, The Fourth Hospital of China Medical University, Shenyang, China; ^3^ Department of Sport Medicine and Joint Surgery, The First Hospital of China Medical University, Shenyang, China

**Keywords:** osteoarthritis, chondrocyte, protein activity inference, single-cell RNA sequencing, senescence

## Abstract

**Background:**

Osteoarthritis (OA) is a prevalent senescence-related disease with substantial joint pain, loss of joint function, and cartilage degeneration. Because of the paucity of single-cell studies of OA and the gene dropout problem of single-cell RNA sequencing, it is difficult to acquire an in-depth understanding of the molecular characteristics of various chondrocyte clusters.

**Methods:**

Here, we aimed to provide new insights into chondrocyte senescence and a rationale for the development of effective intervention strategies for OA by using published single-cell RNA-sequencing data sets and the metaVIPER algorithm (Virtual Inference of Protein activity by Enriched Regulon). This algorithm was employed to present a proteome catalog of 62,449 chondrocytes from the cartilage of healthy individuals and OA patients at single-cell resolution. Furthermore, histopathologic analysis was carried out in cartilage samples from clinical patients and experimental mouse models of OA to validate above results.

**Results:**

We identified 16 protein-activity-based chondrocyte clusters as well as the underlying master regulators in each cluster. By assessing the enrichment score of each cluster in bulk RNA-sequencing data, followed by gene-set variation analysis, we preliminarily identified a novel subpopulation of chondrocytes (cluster 3). This clinically relevant cluster was predicted to be the main chondrocyte cluster responsible for maintaining cellular homeostasis and anti-senescence. Specifically, we uncovered a set of the key leading-edge proteins of cluster 3 by validating the robustness of the above results using another human chondrocyte single-cell RNA-sequencing data set, consisting of 24,675 chondrocytes. Furthermore, cartilage samples from clinical patients and experimental mouse models of OA were used to evaluate the expression patterns of these leading-edge proteins, and the results indicated that NDRG2, TSPYL2, JMJD6 and HMGB2 are closely associated with OA pathogenesis and might play critical roles in modulating cellular homeostasis and anti-senescence in chondrocytes.

**Conclusion:**

Our study revealed a novel subpopulation of chondrocytes that are critical for anti-progression of OA and the corresponding master regulator proteins, which might serve as therapeutic targets in OA.

## Introduction

Osteoarthritis (OA) is a prevalent condition associated with substantial joint pain and loss of function and is predicted to become the main cause of disability among people > 40 years old by 2040 (1). Although the pathogenesis of OA has been studied extensively, there is currently no efficient treatment that can arrest or reverse OA progression. Although several factors, such as body weight, genetic heritage, mechanical stress, trauma, and metabolism, are associated with OA progression, the alterations in cell signaling and homeostasis that occur in chondrocytes are not yet fully known (2, 3). As age is increasingly recognized to be closely related to cellular senescence and OA (4), and chondrocytes (a unique resident cell type in the articular cartilage) are primarily thought to play a major role in maintaining cartilage homeostasis, it is reasonable to hypothesize that chondrocyte senescence is a critical component of OA pathology.

Cellular senescence is a stress response that is primarily designed to eliminate damaged cells and facilitate tissue regeneration. However, with aging or persistent stress, senescence may directly induce the pathogenesis of diseases through stable cell-cycle arrest, DNA damage, and impaired tissue regeneration (5). Multiple signaling pathways involved in cellular senescence, such as the DNA repair, NF-kB, and mTOR signaling pathways, have been described in detail (6–8). Chondrocyte senescence has been increasingly implicated in inflammatory responses, mitochondrial function, and cell cycle, all of which are involved in OA (9–11). The role of senescence in OA has been studied in recent years. For example, previous studies have demonstrated that the proportion of chondrocytes expressing the senescence marker SA-β-Gal increases with the degree of articular lesions in the knee (12). However, since the phenotypic presentation of senescence is highly heterogeneous among tissue types, the underlying mechanism of senescence in OA remains unclear.

Single-cell RNA sequencing (scRNA-seq) has recently emerged as a novel and powerful technology that allows the assessment of the transcriptional states and fundamental biological properties of cell populations at single-cell resolution. scRNA-seq has helped us to better understand many degenerative diseases. For example, Fuchou Tang et al. have preliminarily identified 7 chondrocyte clusters during OA progression by profiling the scRNA-seq data of 1464 chondrocytes from patients with different stages of OA (13). Another scRNA-seq study has reported the profiling of the articular cartilage chondrocytes from healthy and injured mouse knee joints and identified 9 chondrocyte clusters with different biological functions (14). Furthermore, it is worth noting that Tamas Kiss et al. have identified senescent cerebromicrovascular endothelial cells in the aged mouse brain *via* scRNA-seq and enrichment analysis (15). However, it is well known that scRNA-seq-generated gene-expression data are extremely sparse, with as many as 80%–90% of unique mRNA molecules left undetected in every single cell, a problem that is known as “gene dropout.” Thus, although scRNA-seq is effective in distinguishing between cellular subpopulations, it cannot be used to detect the subtle differences between cells or characterize sophisticated biological mechanisms or critical genes, such as transcription factors. This limitation becomes more pronounced in the application of scRNA-seq on the cartilage tissue because the heterogeneity between healthy and OA chondrocytes is not as much as that between different cell types, such as chondrocytes and synoviocytes. To partly compensate for this limitation of scRNA-seq, Andrea Califano et al. have developed the metaVIPER algorithm (Virtual Inference of Protein activity by Enriched Regulon), which can infer accurate and quantitative assessment of protein activity from scRNA-seq data (16). Protein-activity–based clustering can help differentiate between subtle subpopulations and master regulator proteins (MRs) that are responsible for key cellular phenotypes. For instance, the application of the metaVIPER algorithm in clear cell renal carcinoma has helped to identify a subpopulation of recurrence-associated renal tumor macrophages (17).

This study primarily aimed to provide new insights into OA pathogenesis and a rationale for the development of effective intervention strategies against OA, by using the scRNA-seq analysis and metaVIPER algorithm. In this study, scRNA-seq data of chondrocytes (accession numbers: GSE169454 and GSE152805) were subjected to VIPER-based scRNA-seq analysis. Consequently, a new subpopulation of chondrocytes that are mainly responsible for maintaining cellular homeostasis and anti-senescence, as well as potential therapeutic targets were identified. Furthermore, we used human cartilage samples and mouse models of OA to assess for the involvement of these predicted factors in the pathogenesis of OA.

## Methods

### Human chondrocyte single-cell RNA-seq data preprocessing and quality control

Two Human chondrocytes 10X Genomics scRNA-seq data (Gene expression UMI count matrices), GSE169454 and GSE152805 were downloaded from Gene Expression Omnibus (GEO) database (https://www.ncbi.nlm.nih.gov/gds) respectively, which is based on the Illumina HiSeq 2500 and Illumina HiSeq 4000 platforms (Illumina, Inc.). GSE169454 was mainly analyzed, while GSE152805 was used to further validation. There were 7 cartilage samples obtained from femoral condyle cartilage in GSE169454, 4 OA samples of which were obtained from 4 patients receiving total knee arthroplasty surgery for OA, and 3 healthy samples of which were obtained from fresh osteochondral allografts discarded following donor plug harvesting during surgical osteochondral allograft implantation and also procured from NDRI (National Disease Research Interchange, Philadelphia, PA). 6 cartilage samples in GSE152805 were harvested from 3 OA patients’ smooth articular surfaces area in lateral platform of tibial (relatively healthy cartilage) and damaged cartilage articular surfaces area in medial platform of tibial (typical degenerative cartilage) respectively. The detailed patient’s information, sample preparation and scRNA-seq process were described in the authors’ publication (18, 19). Seurat R package (version 4.1.1) (20) was used to data quality control and further analyze the scRNA-seq data. Gene expression UMI count matrices for each sample were read into Seurat respectively by CreateSeuratObject function with the default parameters. Subsequently, cells were removed that had either fewer than 1000 UMIs, over 8000 or below 300 expressed genes, over 5% UMIs derived from mitochondrial genome, or log10 UMIs of per gene lower than 0.8 as a further quality control. After this quality control step, Sctransform function, using regularized negative binomial regression, was used to normalize the data set based on the 3000 most variable genes for each sample. Next, FindIntegration Anchors and IntegrateData function with the default parameters, as suggested by Seurat pipeline, were used to combine scRNA-seq data across all samples.

### Human chondrocyte single-cell RNA-seq data clustering and identification of marker genes

After data integration, the dataset was reduced by principal component analysis (PCA), and the number of principal components was estimated by an Elbow plot for downstream analysis. We then performed a non-linear dimensional reduction *via* uniform manifold approximation and projection (UMAP). Subsequently, total cell clustering was performed using Seurat’s FindNeighbors and FindClusters functions based on the Euclidean distance in PCA space and Louvain algorithm. Marker genes per cluster were determined by Seurat’s FindAllMarkers function with the ‘MAST’ test option. All provided *p*-values were adjusted by bonferroni correction. The results of marker genes were visualized by heatmap and dotplot using the pheatmap (version 1.0.12) and Seurat R package.

### GO and KEGG enrichment analysis

Gene ontology (GO) and Kyoto Encyclopedia of Genes and Genomes (KEGG) pathway enrichment analyses were performed using the ClusterProfiler R package (version 4.2.2) (21). *p*−value <0.05 (adjusted by bonferroni correction) was set as the cutoff criterion for the functional enrichment analysis. The results were visualized by dotplot using the ggplot2 package (version 3.3.6).

### Protein activity inference for single-cell RNA-seq data

After quality control, the human chondrocyte scRNA-seq data (Gene Expression UMI count matrices) was implemented *via* the Seurat Sctransform algorithm and integration. Then, we performed PCA and UMAP to reduce dimensionality of dataset. To generate a stable regulatory network, initial unsupervised clustering was performed in a resolution-optimized louvain algorithm.

First, to select an optimum resolution value, Seurat Louvain clustering is performed with different resolution values ranging from 0.01 to 1.0. For each resolution value ranging from 0.01 to 1.0, silhouette score is computed with correlation distance metric. This procedure is repeated for 100 random samples to select the resolution value with maximizes mean silhouette score as the optimal resolution to perform initial Clustering For each cluster subsequently, 250 metaCells per cluster were computed by pooling cells that are close together in either gene expression. PISCES uses a simple K-nearest-neighbors approach to pool cells, then sums reads across neighbors and re-normalizing. Next, the metaCells file of each cluster was subjected to ARACNe algorithm to generate a regulatory network in a 100 bootstrap iterations way using 1785 transcription factors (genes annotated in gene ontology molecular function database as GO:0003700, ‘‘transcription factor activity,’’ or as GO:0003677, ‘‘DNA binding’’ and GO:0030528, ‘‘transcription regulator activity,’’ or as GO:0003677 and GO:0045449, ‘‘regulation of transcription’’), 668 transcriptional cofactors (a manually curated list, not overlapping with the transcription factor list, built upon genes annotated as GO:0003712, ‘‘transcription cofactor activity’’ or GO:0030528 or GO:0045449), 3455 signaling pathway related genes (annotated in GO biological process database as GO:0007165, ‘‘signal transduction’’ and in GO cellular component database as GO:0005622, ‘‘intracellular’’ or GO:0005886, ‘‘plasma membrane’’), and 3620 surface markers (annotated as GO:0005886 or as GO:0009986, ‘‘cell surface’’). The detailed workflow for ARACNe can be downloaded from Github (https://github.com/califano-lab/ARACNe-AP). Each gene set is run respectively. Once a set of regulons has been inferred for each group of regulators, the results are combined into a network.The Parameters are set as follows: zero DPI, MI (Mutual Information) *p*-value threshold of 10. Based on cluster-specific regulatory networks, protein activity was inferred for scRNA-seq data by a final metaVIPER algorithm run. Then the VIPER-inferred protein activity matrices can be re-clustered also in a resolution-optimized louvain algorithm way after RunPCA and RunUMAP function. The master regulator of each VIPER cluster was identified using bootstrapped t test (100 bootstraps). Then, the top MRs for each cluster were used for downstream analysis. The detailed workflow and statistical method were described in the authors’ publication (16, 22). The gene list used to generate regulatory network can be downloaded from Github (https://github.com/califano-lab/PISCES).

### Bulk RNA-seq data processing

The dataset GSE114007 [deposited by Fisch et al. ((23))], which contains 18 healthy and 20 OA human knee cartilage samples, was downloaded from the GEO database (http://www.ncbi.nlm.nih.gov/geo/). Subsequently, the mRNA count data were subjected to EdgeR (version 3.36.0) package for normalization, counts per million (CPM) and log2CPM transform (24). The microarray data probe was transformed to gene symbols with Bioconductor Annotation Data software packages (version 1.20.0) (25). In the case that 1 single gene symbol was captured by several probes, the final gene expression level was calculated from the average value of those probes. When one probe was mapped to multiple gene sets, information about the probe was deleted. The differential expressed genes (DEGs) between OA and healthy samples were identified through the limma-voom (26). A | Log2(fold change) |>1 and adjusted *p*-value of < 0.05 were set as the cut-off criteria. To control the false discovery rate, adjusted *p*-values were computed for multiple testing corrections of the raw *p*-value through the benjamini-hochberg (BH) method. The detailed patient’s information, sample preparation and RNA sequencing process were described in the authors’ publication (23).

### Gene set enrichment analysis

In order to explore the relationship between MRs of VIPER clusters and OA progression, gene set enrichment analysis (GSEA) was performed. Enrichment of the top100 MRs in each of the VIPER clusters in the DEGs list ranked by fold change of gene expression between OA group and healthy group from bulk-RNASeq was computed respectively by clusterProfiler R package (version 4.2.2) (21). |NES|>1 and p <0.05 were considered to be enrichment significant. MRs in the leading edge of the enrichment was used for further analysis.

### Gene set variation analysis

In order to explore the relationship between VIPER clusters and senescence associated signaling pathways, GSVA enrichment score of the “DNA Repair signaling pathway”, “NF-kB signaling pathway”, “mTOR signaling pathway”, “Mitochondria pathway”, “Biological Oxidations” and “IGF-1/AKT signaling pathway” in each chondrocyte of scRNA-seq data were calculated using the “GSVA” R package (version 1.40.1) (27). The GSVA enrichment score variation in different group (healthy and OA) and VIPER clusters (1-16 clusters) were visualized by violin plot.

### Real-time reverse transcription-polymerase chain reaction

For RNA extaction, about 1g of cartilage was frozen in liquid nitrogen and crushed, then homogenized in Trizol at a concentration of 1g tissue per 10ml Trizol (Invitrogen) followed by incubation at 4°C for 2 hours. 0.2 volumes of chloroform was added, vortexed for 20s, and centrifuged at 14000 rpm for 15 minutes at 4°C. The colorless upper aqueous phase was removed gently into a new tube, and mixed with an equal volume of 100% isopropanol followed by incubation at room temperature for 10 minutes. The mixture was centrifuged at 14000 rpm for 20min at 4°C, and the RNA formed a gel-like pellet on the bottom of the tube. After cleanup using 75% ethanol in RNase-free water, the RNA was dried for 5 minutes.

Single-stranded cDNA was synthesized from purified RNA using a RevertAid first-strand cDNA synthesis kit (Thermo Fisher Scientific, USA) in accordance with the manufacturer’s instructions. Each cycle consisted 30s for denaturation at 95°C, 30s of annealing at 56.5, 57, 57.5 or 58°C, and 30s for extension at 72°C, with total of 35 cycles. The reverse transcription was performed at 37° C for 15 minutes while the heat inactivation of the reverse transcriptase was performed at 85° C for 5 seconds. cDNA was used for real-time PCR analysis using a SYBR^®^ Premix Ex TaqTM kit (Takara Bio, China) on an ABI 7500 Fast Real-Time PCR system (Applied Biosystems, USA) according to the manufacturer’s instructions. The primer sequences are listed in **Table S1**. All samples were analyzed in triplicate. The mRNA value for the target gene was determined using the 2^−ΔΔCt^ method.

### Human articular cartilage samples and animal OA model

Clinical sample collection in this study was reviewed and approved by the Institutional Review Board (IRB) of the First Hospital of China Medical University (EC-2021-HS-004). All patients involved in this study gave informed consent. 10 pairs of smooth and damaged cartilage samples (condyles of femur) were obtained from 10 patients with medial compartment OA who had just undergone total knee arthroplasty (Total cartilage samples = 20). The difference between the smooth (relatively healthy area) and damaged (severely damaged area) articular surfaces was distinguished using the Mankin scoring system (28). Detailed patients’ information is listed in **Table S2**. The animal experiments were carried out with the approval of the Animal Ethical Committee of China Medical University (CMU2021029). All mice were housed with a 12 h light/dark cycle at a constant room temperature (25° C) with free access to food and water. The experimental mice were anesthetized with 3% isoflurane. Experimental OA was induced in 12-week-old male mice (C57BL/6J) by destabilization of the medial meniscus (DMM) surgery as described by Glasson et al. (29). The medial meniscus tibial ligament was exposed without sectioning in sham surgery. Mice were sacrificed 8 weeks after DMM or sham surgery and subjected to histological analyses. After surgical harvesting, the samples from patient or mice were immediately stored at -80° C for mRNA extraction or fixed in 4% paraformaldehyde for histological and immunohistochemical analyses. All human cartilage samples were used for both real-time reverse transcription-polymerase chain reaction and immunohistochemistry. All cartilage samples from OA animal model and aged mouse were used for immunohistochemistry.

### Histopathologic analysis

Human knee articular samples and mice knee joints were prepared and fixed in 4% paraformaldehyde. Then, the samples were decalcified in 10% EDTA for 21 days and embedded in paraffin. Tissue sections (5μm) were stained with safranin O/fast green following standard protocols to determine cartilage degradation under light microscopic examination. The Osteoarthritis Research Society International (OARSI) scoring system was used to assess joint cartilage degeneration. Because both the tibial and femoral cartilages were assessed in the present study, the maximum OARSI score was 12. Three independent investigators who were blinded to the experimental groups performed the scoring. Immunohistochemistry was further performed to analyze the protein expression in histological sections of human knee articular samples and mice knee joints. Primary antibodies were used at 1:100-1:200 dilutions and incubated overnight at 4° C. Then, the sections were incubated with a biotinylated secondary antibody. The reaction was developed using a DAB kit (BD Bioscience, Franklin Lakes, NJ, USA). Primary antibodies against NDRG2 (Proteintech, Cat No. 12015-1-AP), WSB1 (Proteintech, Cat No. 11666-1-AP; Santa Cruz, Cat No. sc-393200), JMJD6 (Proteintech, Cat No. 16476-1-AP), TSPYL2 (Abcam, Cat No. ab240596), HMGB2(Proteintech, Cat No. 14597-1-AP) and PPP1R15A (Proteintech, Cat No. 10449-1-AP) were used at 1:100-1:200 dilutions. The expression levels were evaluated by calculating the percentage of immunopositive cells. Stained positive were defined as the cells are reflective of greater than 10-fold intensity above the background (ImageJ, scanning densitometry). For each joint, the percentage of positive cells was counted on 5 fields and the median percentage was representative for each mouse.

### Statistical analysis

All statistical analyses were performed using R packages. Student’s *t* test was used for comparing two groups. A normality test was applied first for continuous variables before any further comparison analyses. Nonparametric Mann–Whitney U test were used when the data were not normally distributed. OARSI grade data are not continuous and do not follow a normal distribution, were analyzed using non-parametric statistical methods. All histology and immunohistochemistry experiments were conducted on at least three independent biological replicates. The n-value indicates the number of human specimens or mice per group. The sample size n required for each group for animal studies to provide sufficient power was determined based on the design of our previous study. No statistical method was used to predetermine clinical sample sizes. A *p*-value < 0.05 was considered statistically significant.

## Results

### Single-cell transcriptome profiling and clustering of chondrocytes from the human cartilage

**Figure 1:**
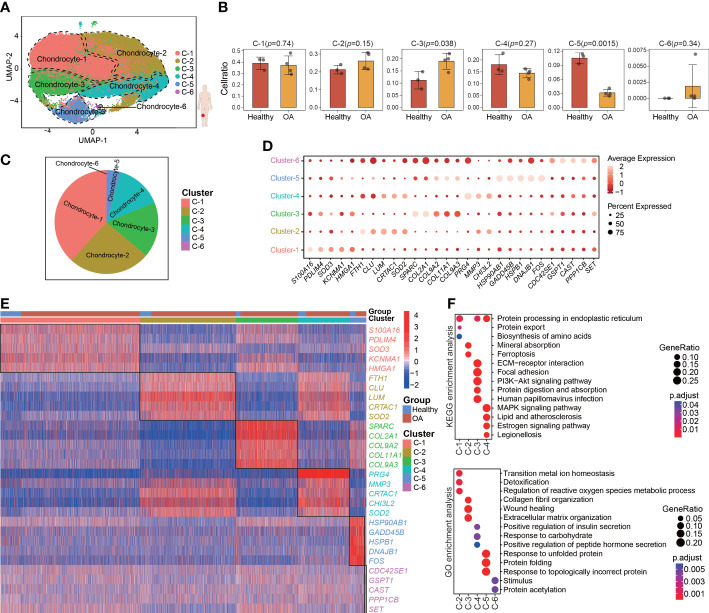
Single-cell Transcriptome profiling and clustering of human cartilage chondrocytes. **(A)** Visualization of umap colored according to cell clusters for 62449 chondrocytes from human OA cartilage single-cell transcriptomes. **(B)** Bar plots showing the comparison of different cell clusters between normal and OA samples. **(C)** Pie chart showing the distribution of different cell clusters. **(D)** Dot plot showing the expression of selected markers (top 5 marker genes, ranked by ‘‘logFC’’) of various cell clusters. **(E)** Heatmap of the scaled expression of top 5 marker genes for each cluster. **(F)** Dot plot showing the significant signaling pathways and biological processes identified by GO and KEGG enrichment analysis. All data are expressed as the mean ± SD. Student’s t test was used for statistical analysis. *p* < 0.05 was considered statistically significant.

To characterize the pathological process of OA at single-cell resolution, we downloaded the GSE169454 dataset from the GEO database. This dataset contains 4 OA and 3 healthy cartilage samples obtained from femoral condyle cartilage. After quality control (**Figures S1A, B**), we retained a total of 62449 cells from 7 individuals (8632 cells from 3 healthy samples and 53817 cells from 4OA samples), which were considered to comprise high-quality data for subsequent analyses through standard seurat process. To investigate chondrocyte heterogeneity in human OA cartilage, after PCA (**Figures S1C, D**) we clustered all the 62449 human chondrocytes by using UMAP, whereby we identified a total of 6 chondrocyte clusters (seurat cluster 1-6) (**Figure 1A**). The chondrocytes in all the samples and groups (healthy and OA) were spread out on the UMAP plot (**Figures S1F, G**). Cellular cluster composition was similar between the healthy and OA human cartilage group (**Figure S1H**). The proportions of seurat cluster 3 and 5 were significantly different between the healthy and OA human cartilage samples, whereas the proportions of seurat clusters 1, 2, 4, and 6 were comparable across all the cartilage samples (**Figures 1B, C**; **S1E**). However, in the UMAP plot, the separation distances among the identified seurat chondrocyte clusters were not well distinguished, consistent with previous reports (13, 18). This suggesting that the chondrocyte heterogeneity isn’t evident between seurat clusters.

We next used differential gene expression analysis to identify marker genes with highly different levels among the seurat clusters (**Figure 1D**; **Table S3**). The heatmap shows the top 5 marker genes that are most highly and uniquely expressed in each seurat cluster, such as *S100A16*, *FTH1*, *SPARC*, *PRG4*, *HSP90AB1*, and *CDC42SE1* (**Figure 1E**). To assess the involvement of each cluster in biological processes and signaling pathways, GO and KEGG analyses were performed using the top 50 marker genes, whereby we identified the distinct physiological functions of the seurat clusters (**Figure 1F**). For example, seurat cluster 3 was significantly enriched in the PI3K-Akt signaling pathway and extracellular matrix organization, whereas seurat cluster 4 was significantly enriched in the MAPK signaling pathway and response to carbohydrate.

### Inference of the protein activity from the scRNA-seq data of chondrocytes from the human cartilage

**Figure 2:**
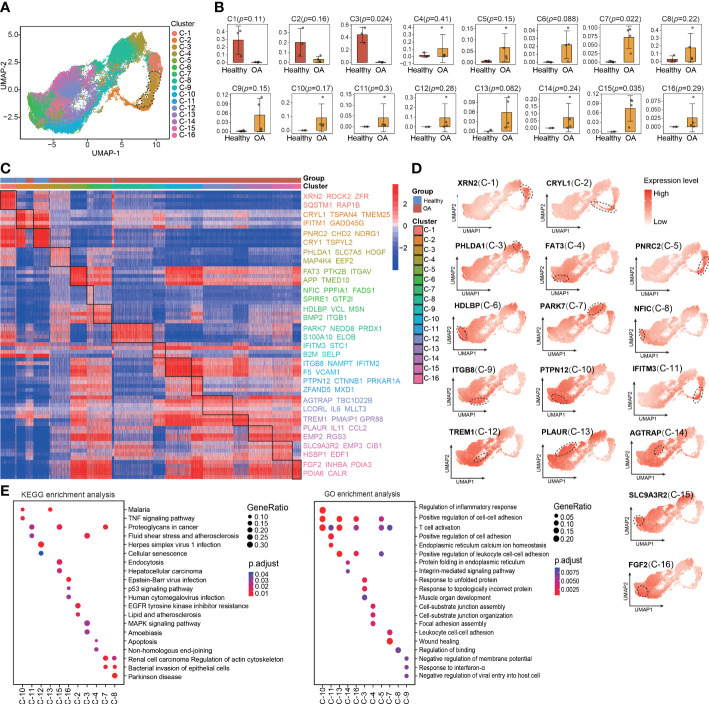
Identification of chondrocyte populations and master regulators from VIPER-Inferred protein activity. **(A)** Visualization of umap colored according to cell clusters for VIPER-Inferred protein activity across all normal and OA samples. **(B)** Bar plots showing the comparison of different cell clusters by VIPER-inferred activity between normal and OA samples. **(C)** Heatmap of the scaled expression of top 5 master regulator proteins of various cell clusters from inferred proteomic data. **(D)** Dot plots showing the top 1 master regulator proteins based on VIPER-inferred protein activity for each viper cluster on the umap. Each dotted line circle in the dot plots represents a viper cluster. Dot colour corresponds to the level of gene expression in each cell. **(E)** Dot plot showing the significant signaling pathways and biological processes identified by GO and KEGG enrichment analysis from inferred proteomic data. All data are expressed as the mean ± SD. Student’s t test was used for statistical analysis. *p* < 0.05 was considered statistically significant.

The main limitation of scRNA-seq is the low signal-to-noise ratio and high dropout rate at the individual gene level (16). To compensate for this limitation, metaVIPER algorithm was invented to transform highly sparse single-cell gene expression matrix to accurate protein activity matrix, which including transcription factors (TFs), co-factors (co-TFs), signaling proteins (SPs), and surface markers (SMs), based on the expression of their downstream regulatory targets. Therefore, we performed VIPER analysis based on metaVIPER R package to assess whether protein-activity–based clustering can help us to identify the chondrocyte clusters that are visually distinct from the other clusters in UMAP and critical for maintaining chondrocyte homeostasis. By using the ARACNe algorithm, the regulatory networks for each of the clusters identified using Seurat were generated. Then, protein activity was inferred from multiple networks by running metaVIPER, resulting in 4331 proteins with successfully inferred activity across all the human chondrocyte samples. Afterward, we re-clustered the protein-activity–based data by using resolution-optimized louvain and thereby identified 16 VIPER clusters (**Figure 2A**). Unlike the RNA expression-based seurat clustering, the UMAP visualization showed that the VIPER cluster 3 was distinctly separated from the other clusters (**Figure 2A**). This suggesting that the phenotype of VIPER cluster 3 is highly different from other VIPER clusters. The proportions of VIPER clusters 3, 7, and 15 were significantly different, whereas those of the other VIPER clusters were comparable, between the healthy and OA human cartilage samples (**Figures 2B**; **S2A, B**).

We next used bootstrapped *t-*test (100 bootstraps) in metaVIPER R package to identify MRs that driving regulators of the differential VIPER cluster as suggested by metaVIPER pipeline (**Table S4**). These MRs represent novel mechanistic drivers of the transcriptional state of these VIPER clusters. The heatmap shows the top 5 MRs in each VIPER cluster, such as XNR2, CRYL1, PNRC2, FAT3, ITGB8, TREM1, and FGF2 (**Figure 2C**). We also found that the MRs of the VIPER cluster more accurately distinguish the difference between the healthy and OA cartilage samples than the marker genes of the seurat cluster (**Figures 2C**; **1E**), which indicating that the MRs of the VIPER cluster are closely related to OA onset and progression. We also observed that the representative proteins of VIPER cluster 3, mapped onto the UMAP plots, were expressed at relatively higher levels than those of most other clusters (**Figure 2D**). Furthermore, KEGG and GO enrichment analyses revealed the involvement of each VIPER cluster in biological processes and signaling pathways. For example, VIPER clusters 10 and 16 were significantly enriched in the TNF and p53 signaling pathways, regulation of inflammatory response, and positive regulation of cell-cell adhesion, whereas VIPER clusters 2 and 3 were significantly enriched in the MAPK signaling pathway, EGFR tyrosine kinase inhibitor resistance, and response to unfolded protein (**Figure 2E**).

### Protein-activity analysis in chondrocytes distinguishes the main subpopulation responsible for modulating cellular homeostasis and anti-senescence

**Figure 3:**
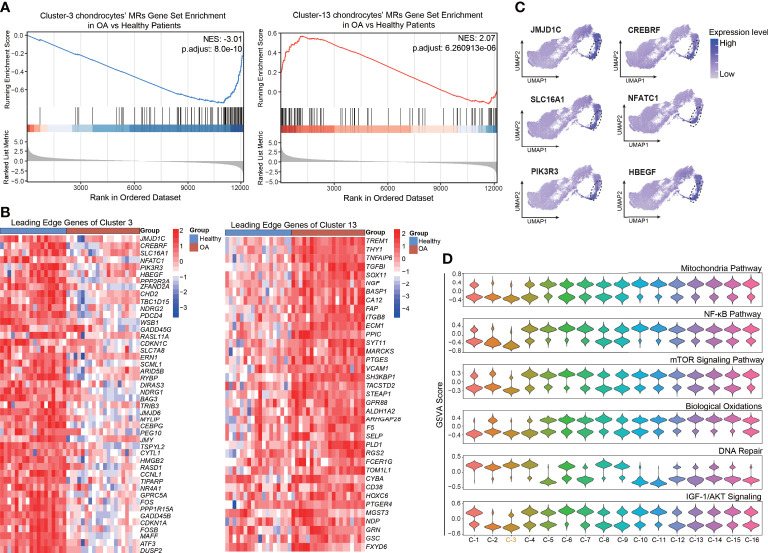
Enrichment of top master regulator proteins of cluster3 and cluster13 from inferred proteomic data in bulk RNA-seq data is associated with OA progression. **(A)** Gene set enrichment analysis (GSEA) of top 100 master regulator proteins of cluster3 and cluster13 from inferred proteomic data in ranked differential expressed genes list of bulkRNA-seq data from 20 patients with OA versus 18 patients with normal cartilage. **(B)** Heatmap of leading-edge protein set of protein activity-based cluster 3 and cluster 13 from GSEA analysis. **(C)** Dot plots showing the top 6 leading-edge protein of cluster 3 based on VIPER-inferred protein activity on the umap. **(D)** Violin plots showing the gene set variation score of DNA Repair signaling pathway, NF-kB signaling pathway, mTOR signaling pathway, Mitochondria pathway, Biological Oxidations, and IGF-1/AKT signaling pathway in various protein activity-based cell clusters.

As described above, we preliminarily identified a set of chondrocyte clusters based on the protein activity and MRs, such as XNR2, CRYL1, and PNRC2, of each VIPER cluster. To reveal the key cluster that maintains chondrocyte homeostasis, we explored the relationship between the VIPER clusters and OA progression. We first downloaded the bulk RNA-seq data set GSE114007 [deposited by Fisch et al. (23)] from the GEO database. This data set contains 18 healthy and 20 OA human knee cartilage tissues and was selected for analysis because of its large sample size and detailed clinical data. Chondrocyte senescence is thought to play a critical role in the pathogenesis of OA, and we found that the mean age of the healthy group in the GSE114007 data set was significantly lower than that of the OA group (36.61 ± 13.46 vs. 66.2 ± 7.16). Therefore, we infer that the difference between the OA and healthy group in this data set not only represents the occurrence of OA but also represents the appearance of tissue aging. After the standard pre-processing of the bulk RNA-seq data, the differentially expressed genes (DEGs) between the OA and healthy patients were identified and ranked by logFC. Then the normalized enrichment score (NES) of the top 100 MRs in each VIPER cluster was computed based on the list of ranked DEGs, with the OA bulk RNA-seq data compared with the healthy-cartilage data. The results of this analysis revealed that VIPER cluster 3 (NES = –3.01, *p* = 8.0e-10) and cluster 13 (NES = 2.07, *p* = 6.26e-06) have the strongest negative and positive enrichment scores in the OA cartilage, respectively, compared with the healthy cartilage (**Figures 3A**; **S2C**). This result suggesting that VIPER cluster 3 and cluster 13 are associated with OA progression and chondrocyte senescence. The leading-edge proteins in enrichment analysis represents the core enriched proteins which may play a critical role in VIPER clusters. And we found that the leading-edge proteins of VIPER cluster 3 and cluster 13 could accurately distinguish between the healthy- and OA-cartilage bulk RNA-seq data (**Figure 3B**; **Table S5**).

To explore the key process that maintains cellular homeostasis and anti-senescence in chondrocytes, we focused on VIPER cluster 3. The expression levels of the top leading-edge proteins of VIPER cluster 3 were visualized on a UMAP dot plot (**Figure 3C**). Since aging is the primary OA risk factor, and senescence of chondrocytes plays a critical role in the pathogenesis of OA (10), we next performed GSVA to assign the estimated activities of senescence-associated signaling pathways, such as the DNA repair, NF-kB, and mTOR signaling pathways, to the individual cells. GSVA is a popular framework to detect subtle pathway activity changes from expression matrix. We first assessed the difference in senescence-associated signaling pathway activity between the healthy and OA groups *via* a protein-activity matrix. The DNA-repair signaling pathway was suppressed in the OA group, consistent with the previous reports that DNA-repair genes are crucial for the maintenance of cell homeostasis (6) (**Figure S2D**). Conversely, the NF-kB (7), mTOR (8), and mitochondria (30) pathways, biological oxidations (31), and the IGF-1/AKT signaling pathway (32), which are closely related to senescence, were activated in the OA group (**Figure S2D**). Consistently, we also observed that the DNA-repair signaling pathway was activated and the NF-kB, mTOR, and mitochondria pathways, biological oxidations, and the IGF-1/AKT signaling pathway were suppressed in the VIPER cluster 3 (**Figure 3D**). No significant changes were observed in the rest of VIPER clusters. Collectively, these results indicate that the VIPER cluster 3 might be the main cluster responsible for modulating chondrocyte homeostasis and anti-senescence. Moreover, as the leading-edge proteins of VIPER cluster 3, which were identified *via* gene set enrichment analysis (GSEA), were strongly associated with the clinical outcomes of OA and were the top MRs in VIPER cluster 3, we assume that these proteins are critical regulators of cartilage homeostasis and may serve as therapeutic targets in OA.

### Validation of the robustness of the leading-edge proteins *via* a VIPER-inferred protein-activity matrix

**Figure 4:**
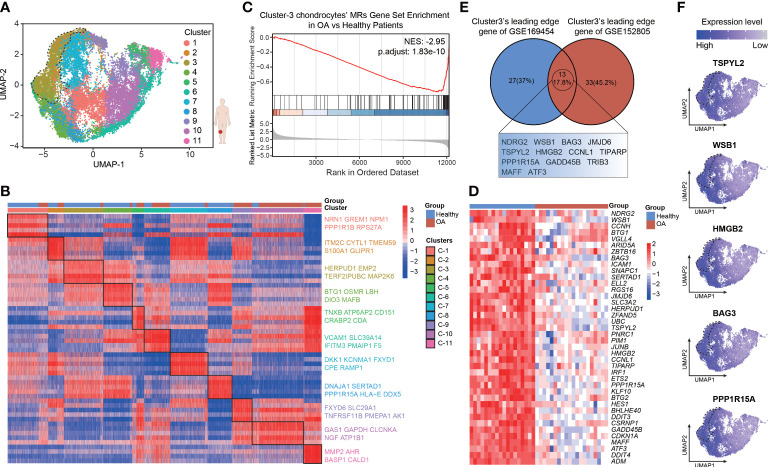
Validation for the robustness of leading-edge proteins based on VIPER-inferred protein activity matrix. **(A)** Visualization of umap colored according to cell clusters for VIPER-Inferred protein activity across 6 cartilage samples. **(B)** Heatmap of the scaled expression of top 5 master regulator proteins of various cell clusters from inferred proteomic data. **(C)** Gene set enrichment analysis (GSEA) of top 100 master regulator proteins of cluster3 from inferred proteomic data in ranked differential expressed genes list of bulkRNA-seq data from 20 patients with OA versus 18 patients with normal cartilage. **(D)** Heatmap of leading-edge protein set of protein activity-based cluster 3 from GSEA analysis. **(E)** Venn plots showing the shared leading-edge proteins of protein activity-based cluster 3 between GSE169454 and GSE152805. **(F)** Dot plots showing the top 5 leading-edge protein of cluster 3 based on VIPER-inferred protein activity on the umap.

To more fully validate the robustness and repeatability of the leading-edge proteins, we analyzed another 10× genomics scRNA-seq data set of human chondrocytes (GSE152805), which contains samples of the smooth articular surface in the lateral tibial platform and damaged articular surface in the medial tibial platform from three OA patients (n = 3 per group). The analytic content is consistent with the above description. After quality control (**Figures S3A, B**), we retained a total of 24675 cells, which comprised high-quality data for PCA (**Figures S3C, D**), UMAP, and clustering analyses (**Figures S4A, C, D**). There are 14349 cells from 3 smooth cartilage samples and 10326 cells from 3 damaged cartilage samples. Cellular cluster composition (**Figure S4B**) and the proportions of all the six seurat clusters (**Figures S4E, F**) were similar between the smooth and damaged human cartilage samples. The heatmap shows the top 5 marker genes most uniquely highly expressed in each seurat cluster, such as *COL9A1*, *NNMT*, *CRTAC1*, *ACTB*, *C2orf82*, and *NFKB1A* (**Figure S4G**; **Table S6**). Then, protein activity was inferred by running metaVIPER, resulting in 4168 proteins with successfully inferred activity across all the human chondrocytes. We re-clustered the data *via* resolution-optimized louvain and thereby identified 11 VIPER clusters (**Figure 4A**). We next used bootstrapped *t*-test (100 bootstraps) to identify the MRs driving the regulators of the differential cluster (**Table S7**). The heatmap shows the top 5 MRs, such as NRN1, ITM2C, HERPUD1, BTG1, TNXB, GAS1, and MMP2, in each VIPER cluster (**Figure 4B**). The NES of the top 100 MRs in each VIPER cluster was computed based on the ranked DEG list of the bulk RNA-seq data, with the OA-cartilage bulk RNA-seq data compared with the healthy-cartilage data. The results of this analysis revealed that VIPER cluster 3 (NES = –2.95, *p* = 1.83e-10) had a higher negative enrichment score in the OA cartilage than in the healthy cartilage (**Figure 4C**). As shown in the heatmap in **Figure 4D**, the leading-edge proteins of VIPER cluster 3 could accurately distinguish between the healthy-cartilage bulk RNA-seq data and the bulk RNA-seq data from typical degenerative cartilage (**Figure 4D**). The above results show that the VIPER cluster 3 in GSE152805 is the key cluster that maintains chondrocyte homeostasis.

It was imperative that the leading-edge analysis found the VIPER cluster 3 in GSE152805 shares many of the same proteins with the VIPER cluster 3 in GSE169454 (13 proteins, 17.8%), such as TSPYL2, WSB1, HMGB2, BAG3 and PPP1R15A (**Figures 4E, F**; **Table S8**). We defined the robust leading-edge proteins that are common between the VIPER cluster 3 in GSE169454 and GSE152805 as the key leading-edge proteins (**Figure 4E**). Interestingly, these common leading-edge proteins, such as WSB1 (33), BAG3 (34), TSPYL2 (35), HMGB2 (36), PPP1R15A (37), and GADD45B (38, 39), have been reported to be intimately correlated with cell senescence. However, we observed that in the UMAP, the separation of the VIPER cluster 3 from the other clusters was not as distinct as in GSE169454 (**Figure 4A**). We infer the reason for this unexpected result is that the cellular difference between the medial and lateral platform cartilage from the same OA patients is not as much as the difference between healthy and OA cartilage from different patients. Taken together, these results suggest that the leading-edge proteins function as critical regulators of chondrocyte homeostasis and anti-senescence and may serve as therapeutic targets in OA.

### The key leading-edge proteins are associated with OA progression

**Figure 5:**
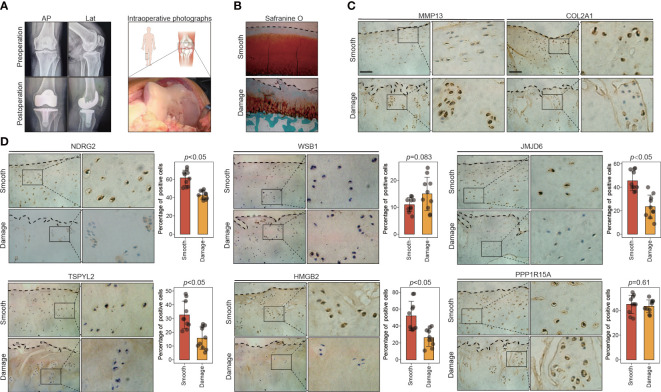
The expression of key leading-edge proteins in the human knee cartilage samples. **(A)** Representative X-rays and intraoperative photographs of clinical cartilage samples (n=10). **(B)** Representative images of safranin O/fast green staining in smooth and damaged cartilage from human knee joints. **(C)** Representative images of immunohistochemical staining with antibodies against MMP13 and COL2A1 in smooth and damaged cartilage from human knee. **(D)** Representative images of immunohistochemical staining with antibodies against NDRG2, WSB1, JMJD6, TSPYL2, HMGB2 and PPP1R15A in smooth and damaged cartilage from human knee. The right panels show the quantification of the immunohistochemical staining. The scale bar represents 50μm. AP, Anteroposterior; Lat, lateral; S, smooth cartilage; D, damaged cartilage. All data are expressed as the mean ± SD. Student’s t test was used for statistical analysis. *p* < 0.05 was considered statistically significant.

To test the validity of the above results, we collected 10 pairs of smooth and damaged cartilage samples from 10 patients with medial compartment OA who had just undergone total knee arthroplasty (**Figure 5A**). Histological differences between the smooth and damaged samples were explored *via* safranin-O/fast-green staining. Severe cartilage loss was observed in the damaged cartilage tissues (reduced safranin-O staining) (**Figure 5B**). Consistent with previous reports, immunostaining results revealed that compared with the levels in the smooth cartilage, the damaged cartilage displayed downregulated COL2A1 and upregulated MMP13, reflecting cartilage degradation (**Figure 5C**). We next explored the expression patterns of the key leading-edge proteins in the smooth and damaged cartilage samples. Among the 13 key leading-edge proteins, the mRNA levels of NDRG2, WSB1, JMJD6, TSPYL2, HMGB2, and PPP1R15A were considerably lower in the damaged cartilage than in the smooth cartilage (**Figure S5**). To test the validity of this finding at the protein level, immunohistochemistry was performed, and the results revealed that compared with the levels in the smooth cartilage, the damaged cartilage displayed decreased protein levels of NDRG2, JMJD6, TSPYL2, and HMGB2 (**Figure 5D**). However, there was no significant difference in the levels of the WSB1 and PPP1R15A proteins between the smooth and damaged cartilage samples (**Figure 5D**). However, these key leading-edge proteins were validated only in human OA samples (smooth and damaged cartilage area) which cannot completely represent pathological differences between healthy and OA condition, such as aging condition. More reliable and comprehensive verification results or therapeutic targets may be obtained from OA and healthy individuals.

**Figure 6:**
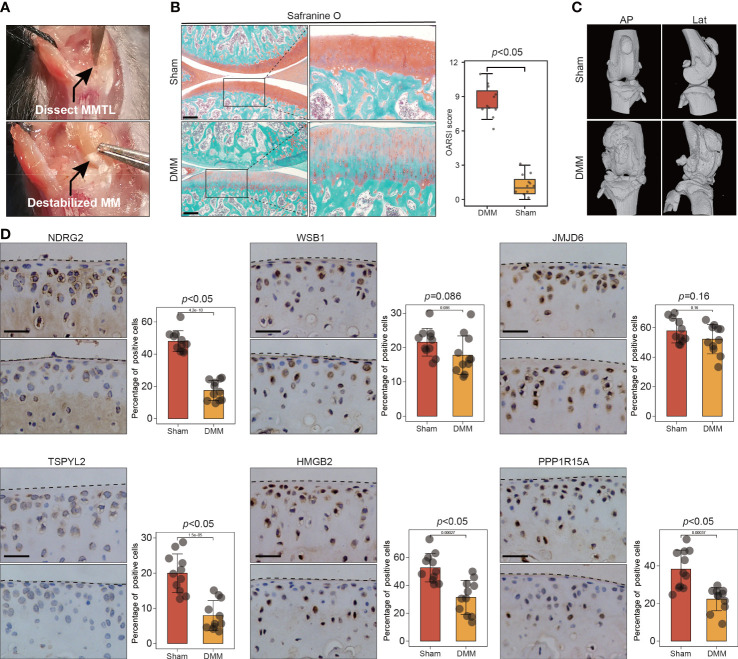
The expression of key leading-edge proteins in the DMM induced mouse model of OA. **(A)** Surgical approach to the right mouse knee joint with transection of the medial meniscotibial ligament. **(B)** Cartilage destruction was determined by safranin O staining and evaluated by OARSI grade. **(C)** representative three-dimensional (3D) reconstruction images of mouse knee joints showing abnormal growth of osteophytes.(n=11 per group). **(D)** Representative images of immunohistochemical staining with antibodies against NDRG2, WSB1, JMJD6, TSPYL2, HMGB2 and PPP1R15A in sham and DMM mouse model of OA. The right panels show the quantification of the immunohistochemical staining. The scale bar represents 50μm. MMLT, medial meniscotibial ligament; DMM, destabilization of the medial meniscus; Sham, sham surgery group; AP, Anteroposterior; Lat, lateral. OARSI grade data are expressed as the interquartile range (from the 25th to the 75th percentiles), with the centerline corresponding to the median. IHC quantification data are expressed as the mean ± SD. Mann–Whitney U test was used for OARSI grade statistical analysis; Student’s t test was used for IHC quantification statistical analysis. *p* < 0.05 was considered statistically significant.

**Figure 7:**
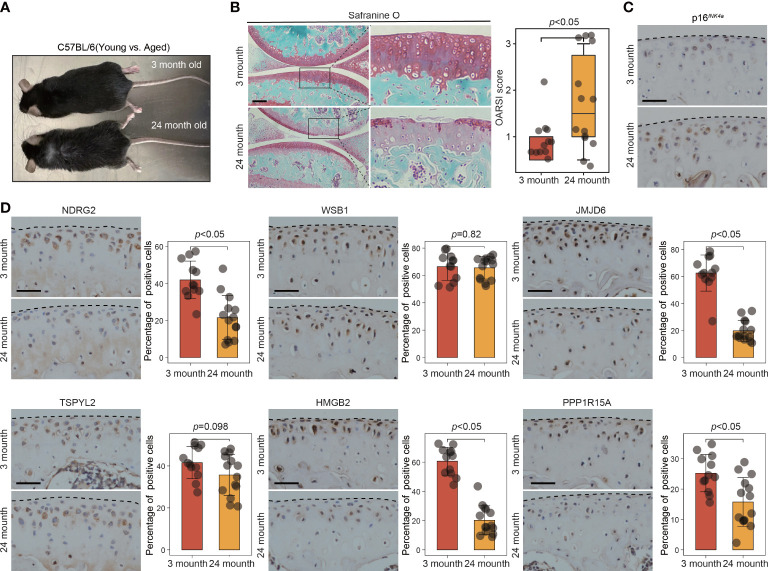
The expression of key leading-edge proteins in aged mouse model of OA. **(A)** Representative photograph of 3-month-old (n=12 per group) and 24-month-old mouse (n=14 per group). **(B)** Cartilage degeneration was determined by safranin O staining and evaluated by OARSI grade. **(C)** Representative images of immunohistochemical staining with antibodies against p16^INK4a^ in 3-month-old and 24-month-old mouse. **(D)** Representative images of immunohistochemical staining with antibodies against NDRG2, WSB1, JMJD6, TSPYL2, HMGB2 and PPP1R15A in 3-month-old and 24-month-old mouse. The right panels show the quantification of the immunohistochemical staining. The scale bar represents 50μm. OARSI grade data are expressed as the interquartile range (from the 25th to the 75th percentiles), with the centerline corresponding to the median. IHC quantification data are expressed as the mean ± SD. Mann–Whitney U test was used for OARSI grade statistical analysis; Student’s t test was used for IHC quantification statistical analysis. *p* < 0.05 was considered statistically significant.

To further validate the involvement of this set of six leading-edge proteins (NDRG2, WSB1, JMJD6, TSPYL2, HMGB2, and PPP1R15A) in OA, we analyzed their expression levels in the C57BL/6 mouse model of OA, which is induced *via* destabilization of the medial meniscus (DMM) surgery (**Figure 6A**). Severe cartilage loss was observed in the damaged cartilage tissues, as determined *via* safranin-O staining and OARSI grade scoring (**Figure 6B**). 3D reconstructions of micro-computed tomography (µCT) scans depicted a gradual increase in mineralized osteophyte formation in the DMM group, compared with the level in the sham group, reflecting OA progression (**Figure 6C**). The results from immunohistochemical staining revealed that compared with the levels in the sham group, the DMM group displayed downregulated NDRG2, TSPYL2, HMGB2, and PPP1R15A (**Figure 6D**). However, there was no significant difference in the levels of the WSB1 and JMJD6 proteins between the DMM and sham groups (**Figure 6D**). To further confirm that these six leading-edge proteins are closely related to senescence, we analyzed their expression levels in aged mouse model of OA (**Figures 7A, B**). Similarly, the expression of NDRG2, HMGB2, and PPP1R15A protein were markedly suppressed in an aged mouse model of knee OA whereas the expression of p16*^INK4a^* was increased in the aging-associated OA cartilage (**Figures 7C, D**).The increased expression of p16*^INK4a^* indicating that increased senescent cells underlie cartilage aging, However, the expression of JMJD6 protein was markedly suppressed in an aged mouse model of knee OA whereas the expression of TSPYL2 protein was not changed, which are different from the results in DMM induced mouse model of OA (**Figure 7D**).

## Discussion

Although several scRNA-seq studies on OA have been reported, the cellular alterations and the disruption of cellular homeostasis in chondrocytes during OA are not yet fully characterized (13, 14, 19, 40). This is probably because first, since chondrocytes are the unique resident cell types in the articular cartilage, the heterogeneity among chondrocytes is not as much as that among the cell types in any other organ. Second, the “gene dropout” problem of the scRNA-seq technology causes gene expression data to be extremely sparse, with as many as 80%–90% of unique mRNA molecules undetected in every single cell. To address this problem, we employed the metaVIPER algorithm in this study, which can accurately infer protein activity through scRNA-seq–generated gene-expression data based on the assumption that only regulons that accurately represent the transcriptional targets of specific proteins in the tissue of interest produce statistically significant enrichment of genes that are differentially expressed in the corresponding tissue (16). Previous studies have shown that metaVIPER is a useful methodology for the analysis of single cell data, and represents a stable repeatability and robustness in protein activity inference (16). Hong Ding et al. conclude that even in the absence of a tissue-matched model, most tissues may be studied virtually without loss of resolution using metaVIPER (16). Aleksandar et al. performed a comprehensive validation of VIPER results using high-parameter spectral flow cytometry. The results found that metaVIPER may potentially outperform antibody-based measurements in terms of both detection and reproducibility, while providing quantitative protein activity inference. By contrast, gene expression-based analyses of scRNA-seq data could not recapitulate flow cytometry results (17). Furthermore, unsupervised clustering analysis of metaVIPER-inferred protein activity efficiently separated single cells with different phenotype (16).

Via scRNA-seq–based gene-expression data and the metaVIPER algorithm, we uncovered the main subpopulation of chondrocytes that are responsible for modulating the cellular homeostasis and anti-senescence in OA. We then identified the MRs and key leading-edge proteins of this chondrocyte subpopulation based on the protein activity, GSEA analysis, and bulk RNA-seq data from human knee cartilage samples. To validate the above results, we used human knee cartilage samples and OA animal models and thereby assessed for an association between the key leading-edge proteins and OA progression.

As described in the results, previous scRNA-seq studies on OA have shown that the separation among the identified chondrocyte clusters in UMAP or t-SNE plots is not distinct enough (13, 14, 19, 40). However, in the present study, the UMAP visualization showed that the VIPER cluster 3 was distinctly separated from the other clusters, and the top MRs of VIPER cluster are more accurate to distinguish the difference between healthy and OA cartilage samples in heatmap compare with top marker genes of the mRNA expression-based cluster. Thus, these data demonstrate that metaVIPER algorithm provide an efficient way to understand the comprehensive atlas of cartilage chondrocytes for which a scRNA-seq generated gene-expression data may be missing. Based on the protein-activity matrix inferred by the metaVIPER algorithm, we identified the MRs of each VIPER cluster. A set of MRs, namely PNRC2, CHD2, NDRG1, CRY1, and TSPYL2, were highly expressed specifically in the VIPER cluster 3. A previous study has demonstrated that CHD2 can effectively promote DNA repair pathway (41). Jinlong Li et al. have reported that CRY1, a circadian-clock protein, can prevent cell senescence by promoting p53 degradation (42). The MRs of each VIPER cluster (**Table S4**) may serve as a reference for future studies.

To assess for the clinical relevance of the VIPER clusters, we used GSEA combined with bulk RNA-seq data and thereby computed the enrichment scores of the top MRs of each cluster in the ranked DEGs of the OA clinical samples, compared with those of the healthy-cartilage samples. We found that the VIPER cluster 3 had a higher negative enrichment score in the OA-cartilage samples than in the healthy-cartilage samples (NES = –3.01, *p* = 8.0e-10). Although several factors, such as body weight, genetic heritage, mechanical stress, trauma, and metabolism are thought to be associated with OA, recent studies have indicated that cellular senescence in the cartilage is the primary driver of OA (10). Notably, the bulk RNA-seq data (GSE114007) used in this study comprised 18 healthy and 20 OA human knee cartilage samples, and the mean age of the healthy group was significantly lower than that of the OA group (36.61 ± 13.46 versus 66.2 ± 7.16). This indicates that the difference between the OA and healthy groups in the bulk RNA seq data represents not only OA pathogenesis but also tissue aging. This allowed us to infer whether the VIPER cluster 3 is the critical chondrocyte subpopulation in the cartilage to modulate cellular homeostasis and anti-senescence. By calculating the gene set variation of each cell in the protein-activity matrix to assess the activity of senescence-associated signaling pathways (the DNA repair, NF-kB, IGF-1/AKT, and mTOR signaling pathways, mitochondria pathway, and biological oxidations), we observed high activity of the DNA repair signaling pathway in VIPER cluster 3. Conversely, we observed low activity of the NF-kB, mTOR, and IGF-1/AKT signaling pathways, mitochondria pathway, and biological oxidations in VIPER cluster 3, suggesting that VIPER cluster 3 is mainly responsible for modulating chondrocyte homeostasis and senescence. For instance, the DNA-repair signaling pathway, with high activity in the VIPER cluster 3, is a fundamental and conserved mechanism responsible for repairing damaged DNA and involved in anti-senescence (43).

To explore the core proteins in VIPER cluster 3 that maintain cellular homeostasis and anti-senescence, we analyzed the leading-edge proteins of VIPER cluster 3, namely JMJD6, CREBRF, SLC16A1, NFATC1, and PIK3R3, *via* GSEA (**Table S5**). Yvan Canitrot et al. have reported that JMJD6 is required for DNA stability through its role in the formation of nucleolar caps (44). PIK3R3, one of the regulatory subunits of phosphoinositide 3-kinase, can inhibit cell senescence through the p53/p21 signaling (45). To validate the robustness of the leading-edge proteins identified in the VIPER-inferred protein-activity matrix, we analyzed another 10× genomics human chondrocyte scRNA-seq data set (GSE152805) through the same process as above. Of note, we also identified a subpopulation of cluster 3 with a stronger negative enrichment score in the OA cartilage than in the healthy cartilage and found that many of the leading-edge proteins of the VIPER cluster 3 of GSE152805 were also among those of the VIPER cluster 3 of GSE169454, such as TSPYL2, WSB1, HMGB2, BAG3, and PPP1R15A. We consider these common robust leading-edge proteins the key leading-edge proteins, which might be directly or indirectly involved in modulating chondrocyte homeostasis and anti-senescence. We tried to test the correlation between leading edge proteins (NDRG2, WSB1, JMJD6, TSPYL2, HMGB2) and chondrocyte senescence marker proteins in protein activity matrix, such as p16, p21, SOX9, COL10A1. However, we found that metaVIPER didn’t detect the protein activity of p16, p21, and there is no significant correlation between leading edge proteins (NDRG2, WSB1, JMJD6, TSPYL2, HMGB2) and chondrocyte senescence marker proteins (SOX9, COL10A1). This may be partly caused by the insufficient sequencing depth and high dropout rate of scRNA-seq. Furthermore, a specific gene expression or not are varies greatly in each cell, which may bias the results of correlation analysis.

To assess for the involvement of these key leading-edge proteins in OA pathogenesis, we evaluated their mRNA expression patterns in clinical cartilage samples *via* reverse transcription–quantitative polymerase reaction. Among the 13 key leading-edge proteins, the mRNA levels of only NDRG2, WSB1, JMJD6, TSPYL2, HMGB2, and PPP1R15A were significantly downregulated in the damaged cartilage samples, compared with the levels in the smooth cartilage samples. Interestingly, these proteins have been reported to be intimately correlated with cell senescence. For instance, WSB1 expression in primary cells promotes ATM ubiquitination, resulting in ATM degradation and helps the bypass of Oncogene-induced senescence (33). Mechanistically, WSB-1 is involved in DNA damage response to regulate cell senescence (46). In response to DNA damage, JMJD6 is required for rDNA stability through its role in nucleolar caps formation (44). Among these 6 proteins, however, only the levels of the NDRG2, JMJD6, TSPYL2, and HMGB2 proteins showed significant changes, based on the immunohistochemical results.

Senescence is also a feature of the chondrocytes in the cartilage of post-traumatic OA (47). Thus, we also evaluated the levels of the NDRG2, WSB1, JMJD6, TSPYL2, HMGB2, and PPP1R15A proteins in a DMM-induced OA mouse model. We observed that only the levels of the NDRG2, TSPYL2, HMGB2, and PPP1R15A proteins were significantly decreased in the cartilage of the OA mouse model. These results are partly different from what we observed in clinical human cartilage samples. We specifically focused on NDRG2, TSPYL2, and HMGB2 since they were identified as the differentially expressed proteins between the healthy- and OA-cartilage samples both in humans and mice.

The IL-1β pro-inflammatory cytokine is usually used to stimulate chondrocytes to undergo OA-like changes *in vitro*. NDRG2 expression is much lower in IL-1β–treated chondrocytes than in untreated cells, and its overexpression by transfection of pcDNA3.1(+)/NDRG2 restores the inflammatory response and ECM degradation in chondrocytes (48). Furthermore, NDRG2 expression level is significantly correlated with astrocyte senescence, and NDRG2 can directly interact with NF-κB and inhibit the nuclear import and DNA-binding activity of the NF-κB p65 subunit in primary astrocytes (49, 50). However, the exact mechanism of NDRG2 in senescence and OA progression deserves further study.

It is well known that the DNA repair signaling pathway is crucial for the maintenance of chondrocyte homeostasis and anti-senescence, especially during aging (51). Previous studies have shown that TSPYL2 plays an important role in DNA damage repair and revealed the molecular mechanisms whereby TSPYL2 regulates SIRT1 and p53 activity upon DNA damage (35, 52). Therefore, we infer that TSPYL2 plays an important role in senescence through regulate DNA repair signaling pathway. However, the mechanism of TSPYL2 in OA progression remains to be further investigated.

Numerous studies have shown that HMGB2 is closely related to senescence. Notably, several studies have found that HMGB2 plays a role in chondrocytes. For instance, previous studies have indicated that chondrocyte senescence is associated with a loss of HMGB2 expression, and HMGB2 promotes chondrocyte proliferation (53, 54). Studies on the molecular mechanism of HMGB2 have shown that HMGB2-mediated genomic reorganization initiates a senescence program and holds the key to the senescence-associated secretory phenotype (36, 55). Furthermore, knocking down HMGB2 suffices for senescence-induced CTCF clustering and for loop reshuffling (36).

This is the first scRNA-seq study to explore chondrocyte senescence and OA pathogenesis based on single-cell protein-activity analysis. However, there are several limitations to this study. First, cell-type annotation of the protein-activity–based clusters, which is a specific challenge in the field of OA research, was not carried out. Second, we did not fully address the accuracy of the metaVIPER-algorithm–inferred protein-activity matrix *via* high dimensional flow cytometry, which was performed in the corresponding publication (17). Thirdly, the author of GSE169454 didn’t mention the 7 cartilage samples were obtained from medial or lateral condylar and GSE152805 scRNA-seq data used in the validation all came from OA samples cannot completely represent pathological change between healthy and OA condition. The above reasons may cause partial information to be obscured in the results of this bioinformatic analysis. Lastly, the present results in our study are based on a bioinformatics analysis and the exact mechanism of NDRG2, WSB1, JMJD6, TSPYL2, HMGB2 in senescence and OA progression deserves further study. We therefore consider these approaches, including the study of osteoarthritis development in surgical and aging models, as the next steps in our quest to understand the role of NDRG2, WSB1, JMJD6, TSPYL2, HMGB2 and PPP1R15A in osteoarthritis and their inner mechanistic links.

## Conclusion

In summary, our study shows for the first time that a novel subpopulation of chondrocytes is mainly responsible for maintaining cellular homeostasis and closely associated with OA progression. We identified protein-activity–based cluster-specific MRs, which highlighted the molecular characteristics of steady-state chondrocytes. A significant association between certain key leading-edge proteins (NDRG2, TSPYL2, and HMGB2) and OA pathogenesis was found. These key proteins are predicted to modulate chondrocyte homeostasis and may serve as therapeutic targets in OA. Additionally, our study suggests that metaVIPER can be effectively applied to other tissue-based scRNA-seq studies.

## Data availability statement

The datasets presented in this study can be found in online repositories. The names of the repository/repositories and accession number(s) can be found in the article/**Supplementary Material**.

## Ethics statement

The studies involving human participants were reviewed and approved by Institutional Review Board (IRB) of the First Hospital of China Medical University (EC-2021-HS-004). The patients/participants provided their written informed consent to participate in this study. The animal study was reviewed and approved by Animal Ethical Committee of China Medical University (CMU2021029).

## Author contributions

GX conceived and designed the study. ZG and ZM performed data analyses and wrote the manuscript. DT-X and LJ-T performed clinical sample collection and animal experiments. All authors contributed to the article and approved the submitted version.

## Supplementary material

Supplementary Figure 1 Quality control of single-cell RNA-seq data and clustering of human chondrocytes. **(A)** Number of UMI, number of features, percent of mitochondrial gene and log10GenesPerUMI distribution of human chondrocytes in each of cartilage sample. **(B)** Visualize the correlation between the number of detected genes and the number of UMI. **(C)** Elbow plot showing the ranking of principle components based on the percentage of variance explained by each one. **(D)** PCA plot of single-cell transcriptomes, colored according to the 7 samples. **(E)** Proportion of different cluster in each clinical sample. **(F)** Visualization of umap colored according to each clinical sample. **(G)** Visualization of umap colored according to the group of clinical samples. **(H)** Visualization of umap colored according to cell clusters for 62449 chondrocytes from human OA cartilage split by OA versus normal label.

Supplementary Figure 2 Identification of chondrocyte populations from VIPER-Inferred protein activity, GSEA and GSVA analysis. **(A)** Pie chart showing the distribution of different cell clusters from VIPER-Inferred protein activity. **(B)** Proportion of different protein activity-based clusters in each clinical sample. **(C)** Gene set enrichment analysis (GSEA) of top 100 master regulator proteins of different clusters from inferred proteomic data in ranked differential expressed genes list of bulkRNA-seq data from 20 patients with OA versus 18 patients with healthy cartilage. Gene list were ranked by the fold change in OA versus healthy patient; the *p* value was computed by GSEA versus gene shuffling the null model with 1,000 permutations. Bar graph represents the leading-edge subset. NES represents normalized enrichment score. **(D)** Box plots showing the comparison of gene set variation score of DNA Repair signaling pathway, NF-kB signaling pathway, mTOR signaling pathway, Mitochondria pathway, Biological Oxidations, and IGF-1/AKT signaling pathway between normal and OA group. GSVA score data are expressed as the the interquartile range (from the 25th to the 75th percentiles), with the centerline corresponding to the median. For GSVA score analysis, statistical analysis was performed using Mann–Whitney U test. *p* < 0.05 was considered statistically significant.

Supplementary Figure 3 Quality control of single-cell RNA-seq data and clustering of human chondrocytes. **(A)** Number of UMI, number of features, percent of mitochondrial gene and log10GenesPerUMI distribution of human chondrocytes in each of cartilage sample. **(B)** Visualize the correlation between the number of detected genes and the number of UMI. **(C)** Elbow plot showing the ranking of principle components based on the percentage of variance explained by each one. **(D)** PCA plot of single-cell transcriptomes, colored according to the 7 samples.

Supplementary Figure 4 Single-cell Transcriptome profiling and clustering of human cartilage chondrocytes. **(A)** Visualization of umap colored according to cell clusters for 24675 chondrocytes from human OA cartilage single-cell transcriptomes. **(B)** Visualization of umap colored according to cell clusters for 24675 chondrocytes from human OA cartilage split by smooth versus damage label. (C) Visualization of umap colored according to each clinical sample. **(D)** Visualization of umap colored according to the group of clinical samples. **(E)** Proportion of different cluster in each clinical sample. **(F)** Bar plots showing the comparison of different cell clusters between smooth and damage group. **(G)** Heatmap of the scaled expression of top 5 marker genes for each cluster. All data are expressed as the mean ± SD. Student’s t test was used for statistical analysis. *p* < 0.05 was considered statistically significant.

Supplementary Figure 5 The mRNA expression levels of key leading-edge proteins in the human knee cartilage samples. Real-time PCR analysis was used to assess the mRNA levels of NDRG2, WSB1, BAG3, JMJD6, TSPYL2, HMGB2, CCNL1, TIPARP, PPP1R15A, GADD45B, TRIB3, MAFF and ATF3 in smooth and damage cartilage from human OA patients (n = 10 per group). All data are expressed as the mean ± SD. Student’s t test was used for statistical analysis. *p* < 0.05 was considered statistically significant.
