# Supplementary figures and images for: Single-cell protein activity analysis reveals a novel subpopulation of chondrocytes and the corresponding key master regulator proteins associated with anti-senescence and OA progression
Source: Front Immunol. 2023 Mar 23;14:1077003. doi: 10.3389/fimmu.2023.1077003 (PMC10077735; doi:10.3389/fimmu.2023.1077003)

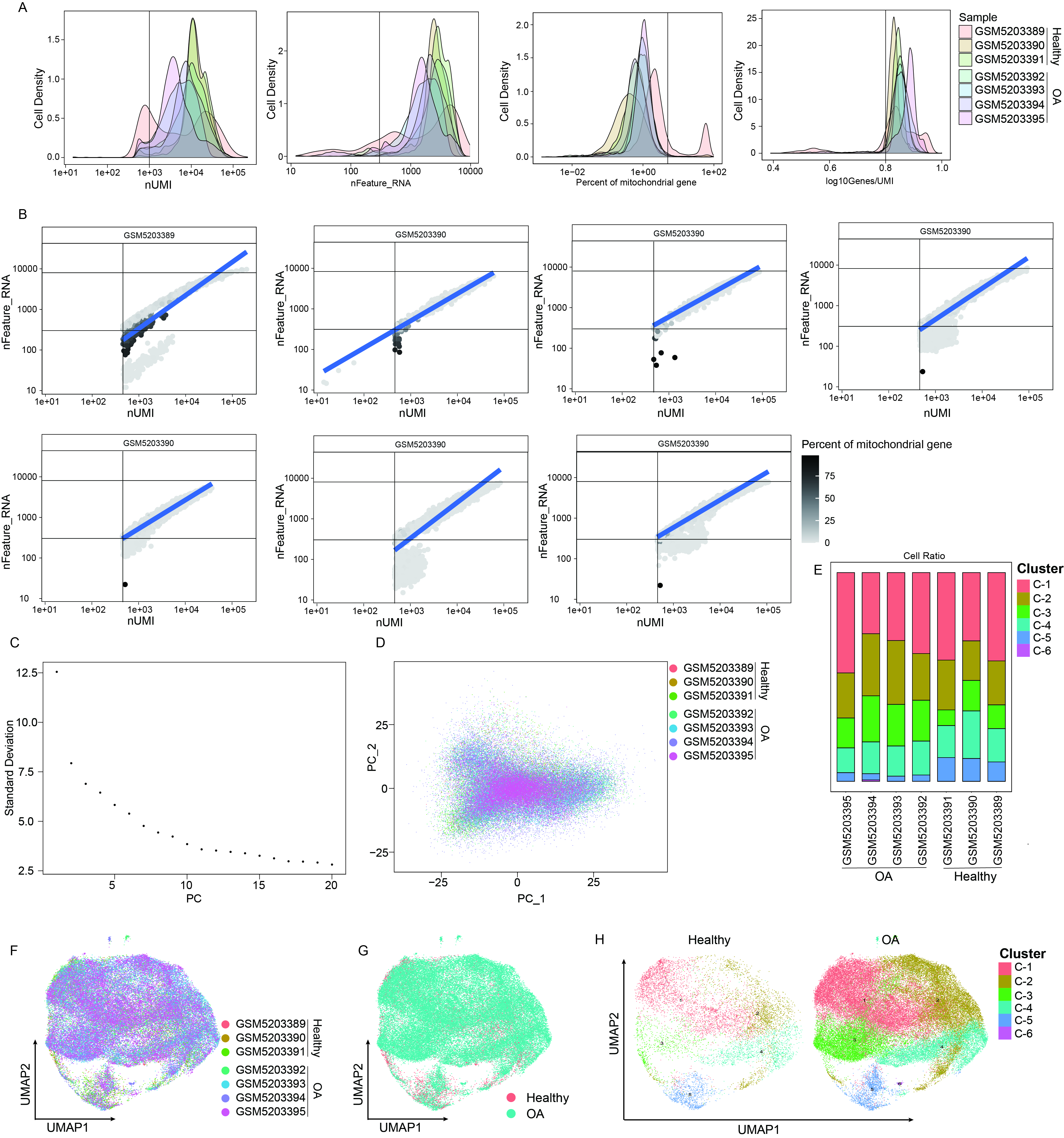

Supplement: Supplementary Figure 1 — Quality control of single-cell RNA-seq data and clustering of human chondrocytes. (A) Number of UMI, number of features, percent of mitochondrial gene and log10GenesPerUMI distribution of human chondrocytes in each of cartilage sample. (B) Visualize the correlation between the number of detected genes and the number of UMI. (C) Elbow plot showing the ranking of principle components based on the percentage of variance explained by each one. (D) PCA plot of single-cell transcriptomes, colored according to the 7 samples. (E) Proportion of different cluster in each clinical sample. (F) Visualization of umap colored according to each clinical sample. (G) Visualization of umap colored according to the group of clinical samples. (H) Visualization of umap colored according to cell clusters for 62449 chondrocytes from human OA cartilage split by OA versus normal label. [file Image_1.tif]

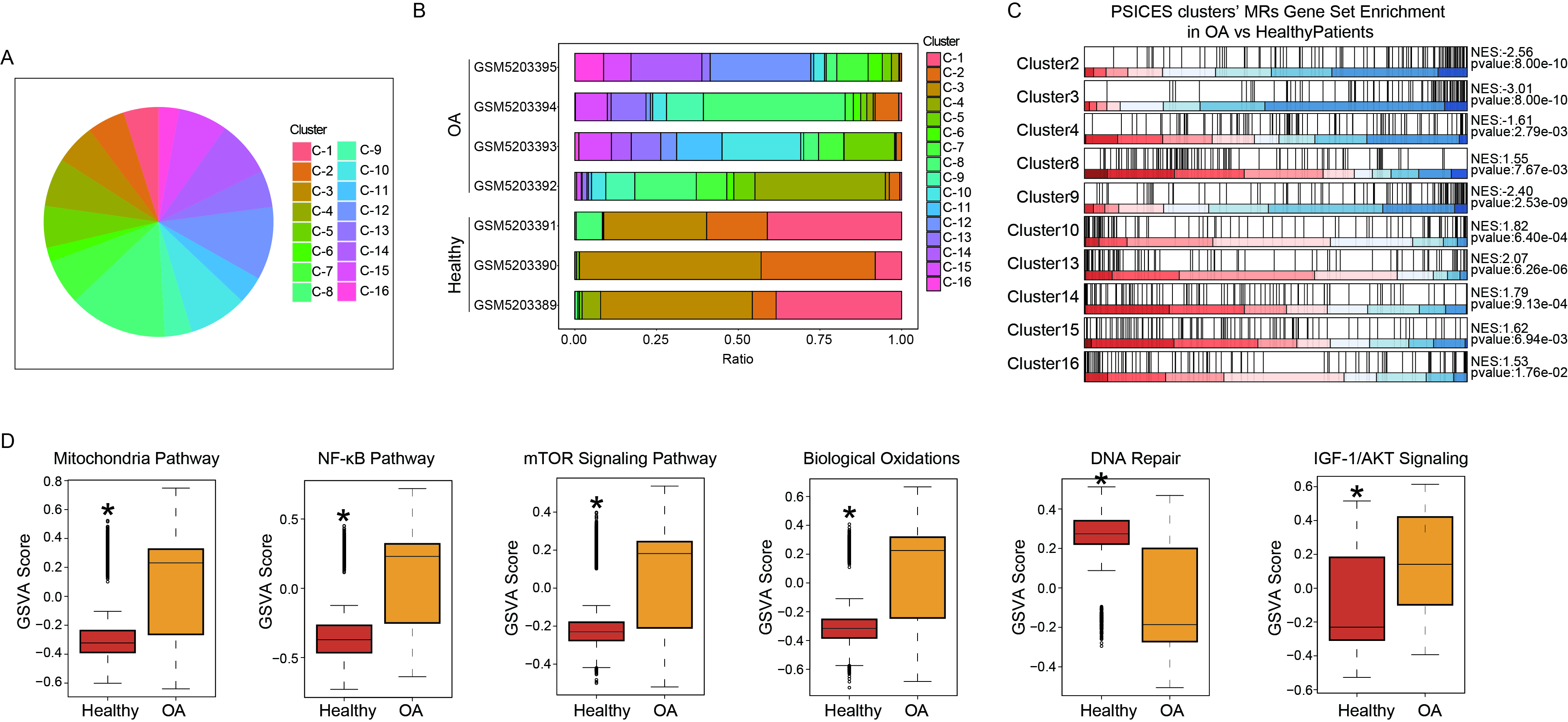

Supplement: Supplementary Figure 2 — Identification of chondrocyte populations from VIPER-Inferred protein activity, GSEA and GSVA analysis. (A) Pie chart showing the distribution of different cell clusters from VIPER-Inferred protein activity. (B) Proportion of different protein activity-based clusters in each clinical sample. (C) Gene set enrichment analysis (GSEA) of top 100 master regulator proteins of different clusters from inferred proteomic data in ranked differential expressed genes list of bulkRNA-seq data from 20 patients with OA versus 18 patients with healthy cartilage. Gene list were ranked by the fold change in OA versus healthy patient; the p value was computed by GSEA versus gene shuffling the null model with 1,000 permutations. Bar graph represents the leading-edge subset. NES represents normalized enrichment score. (D) Box plots showing the comparison of gene set variation score of DNA Repair signaling pathway, NF-kB signaling pathway, mTOR signaling pathway, Mitochondria pathway, Biological Oxidations, and IGF-1/AKT signaling pathway between normal and OA group. GSVA score data are expressed as the the interquartile range (from the 25th to the 75th percentiles), with the centerline corresponding to the median. For GSVA score analysis, statistical analysis was performed using Mann–Whitney U test. p < 0.05 was considered statistically significant. [file Image_2.tif]

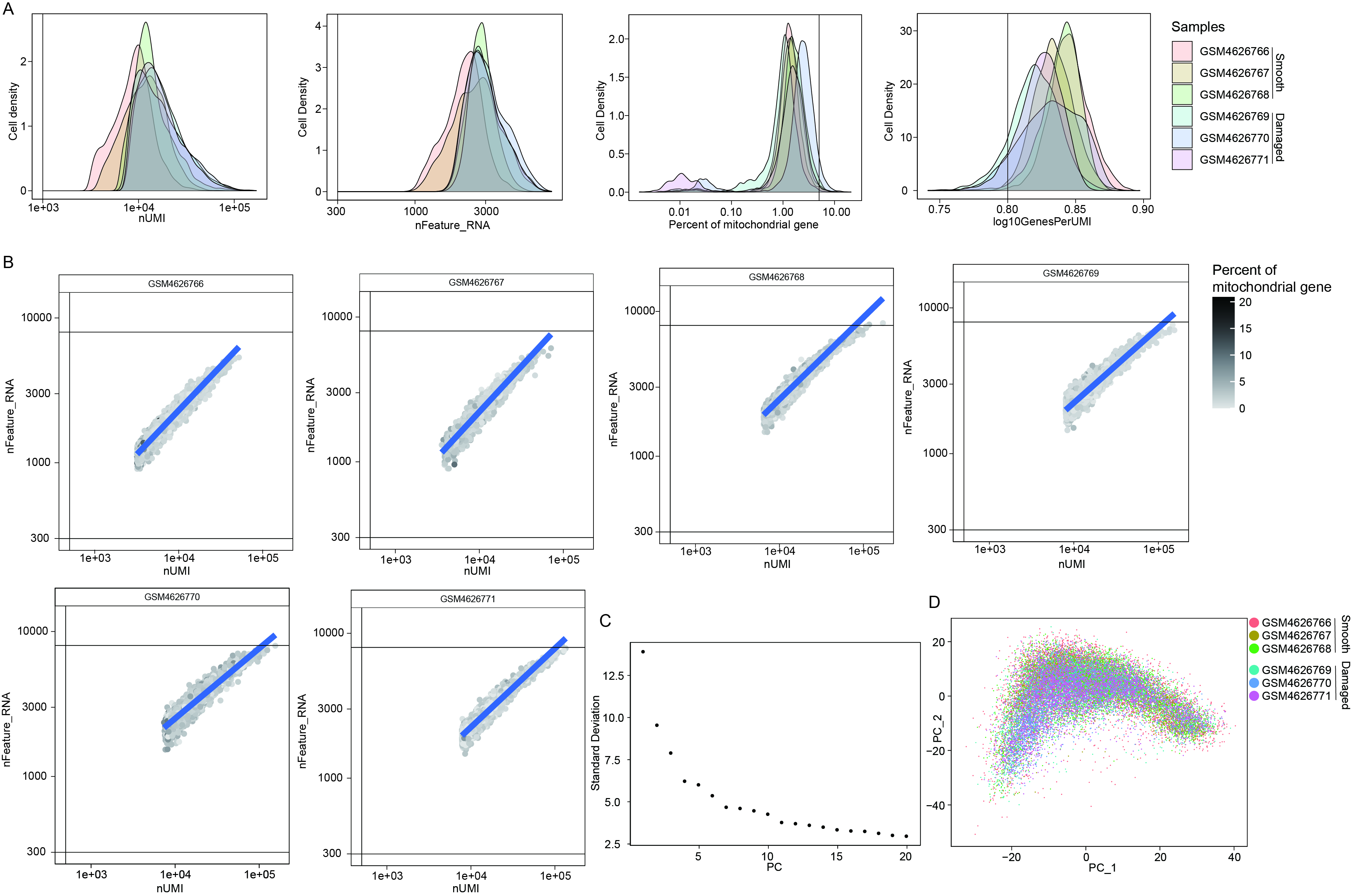

Supplement: Supplementary Figure 3 — Quality control of single-cell RNA-seq data and clustering of human chondrocytes. (A) Number of UMI, number of features, percent of mitochondrial gene and log10GenesPerUMI distribution of human chondrocytes in each of cartilage sample. (B) Visualize the correlation between the number of detected genes and the number of UMI. (C) Elbow plot showing the ranking of principle components based on the percentage of variance explained by each one. (D) PCA plot of single-cell transcriptomes, colored according to the 7 samples. [file Image_3.tif]

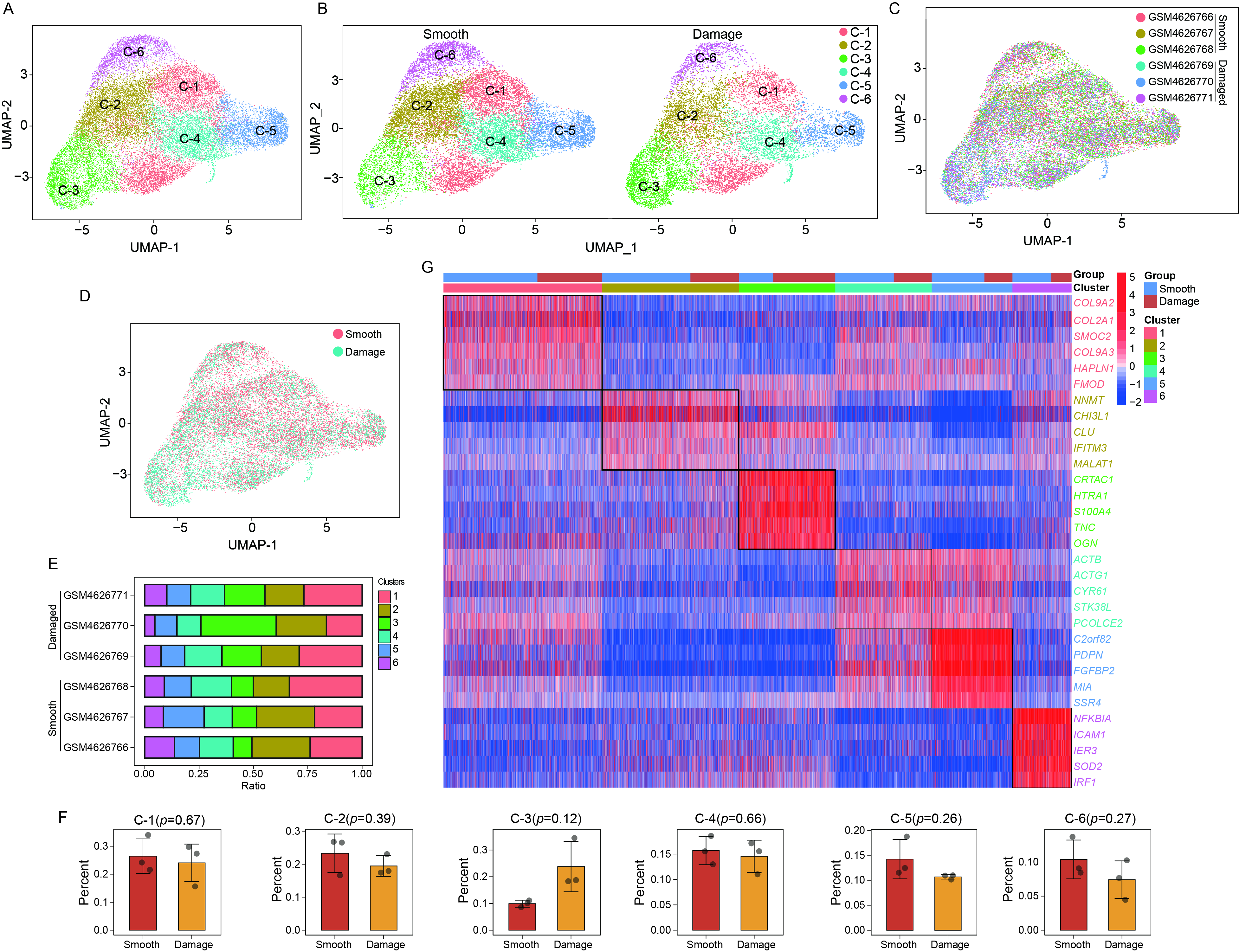

Supplement: Supplementary Figure 4 — Single-cell Transcriptome profiling and clustering of human cartilage chondrocytes. (A) Visualization of umap colored according to cell clusters for 24675 chondrocytes from human OA cartilage single-cell transcriptomes. (B) Visualization of umap colored according to cell clusters for 24675 chondrocytes from human OA cartilage split by smooth versus damage label. (C) Visualization of umap colored according to each clinical sample. (D) Visualization of umap colored according to the group of clinical samples. (E) Proportion of different cluster in each clinical sample. (F) Bar plots showing the comparison of different cell clusters between smooth and damage group. (G) Heatmap of the scaled expression of top 5 marker genes for each cluster. All data are expressed as the mean ± SD. Student’s t test was used for statistical analysis. p < 0.05 was considered statistically significant. [file Image_4.tif]

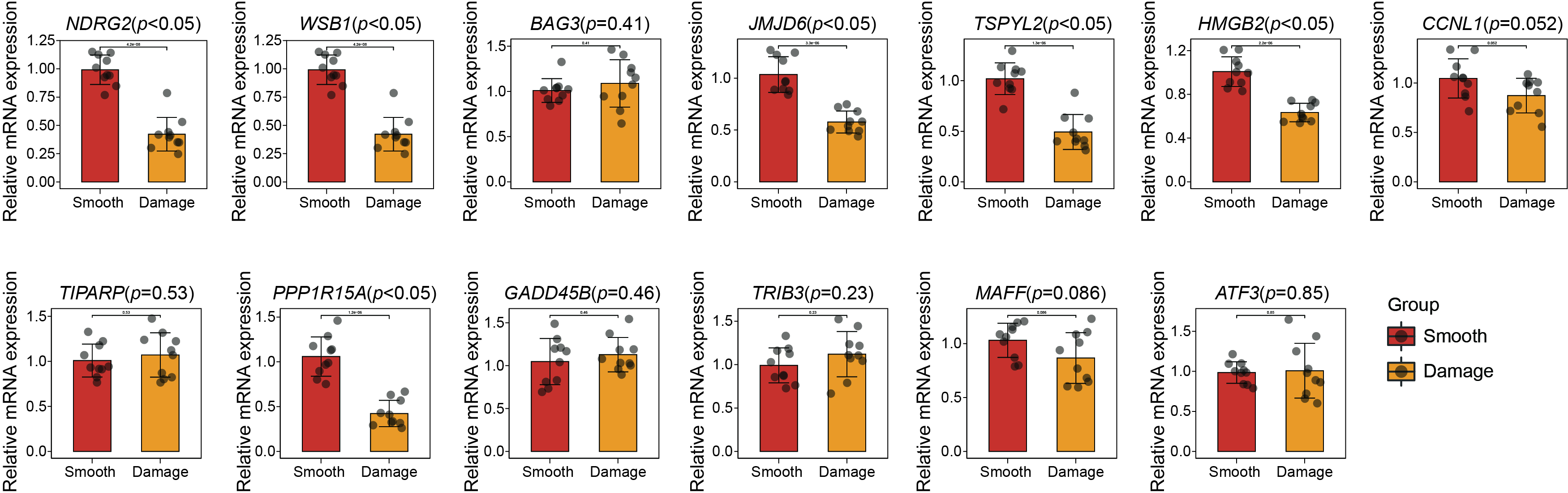

Supplement: Supplementary Figure 5 — The mRNA expression levels of key leading-edge proteins in the human knee cartilage samples. Real-time PCR analysis was used to assess the mRNA levels of NDRG2, WSB1, BAG3, JMJD6, TSPYL2, HMGB2, CCNL1, TIPARP, PPP1R15A, GADD45B, TRIB3, MAFF and ATF3 in smooth and damage cartilage from human OA patients (n = 10 per group). All data are expressed as the mean ± SD. Student’s t test was used for statistical analysis. p < 0.05 was considered statistically significant. [file Image_5.tif]
