# Supplementary material for: Single-cell protein activity analysis reveals a novel subpopulation of chondrocytes and the corresponding key master regulator proteins associated with anti-senescence and OA progression
Source: Front Immunol. 2023 Mar 23;14:1077003. doi: 10.3389/fimmu.2023.1077003 (PMC10077735; doi:10.3389/fimmu.2023.1077003)
Supplement: Supplementary file 6 [file Table_1.docx]

**Table S1 Primer sequence used in Real-Time reverse transcription-polymerase chain reaction**

| **Gene** | **Primer sequence** |
| --- | --- |
| *NDRG2* | Forward primer: 5’- GGATTCATGGCGGAGCTGCAGGAGG-3’  Reverse primer: 5’- GGATTCATGGCGGAGCTGCAGGAGG-3’ |
| *WSB1* | Forward primer: 5’- CGTACTATAGGTGAACTTTTAGCTCCT -3’  Reverse primer: 5’- CCAAAGGAAAACTGCTTTACTGG-3’ |
| *BAG3* | Forward primer: 5’- TGGGAGATCAAGATCGACCC -3’  Reverse primer: 5’- GGGCCATTGGCAGAGGATG -3’ |
| *JMJD6* | Forward primer: 5’- GATCCAGACTCGCACTGGAC -3’  Reverse primer: 5’- CCCGAGGTCAGAGGTTTGTT-3-3’ |
| *TSPYL2* | Forward primer: 5’- AGGCACTGGAGGATATTCAG -3’  Reverse primer: 5’- GAAGGGTCTTCGCATCTGGAT -3’ |
| *HMGB2* | Forward primer: 5’- AAGAGCGACAAAGCTCGTTATG -3’  Reverse primer: 5’- GCAGTATCTCCAATAGACAGGC -3’ |
| *CCNL1* | Forward primer: 5’- AAGAAGCAGGTCCCGCAGT -3’  Reverse primer: 5’- TGGCTGCATCTGAGTGATCC -3’ |
| *TIPARP* | Forward primer: 5’- AGAACGAGTGGTTCCAATCCA -3’  Reverse primer: 5’- TGGGTGCAAAAGATCAGTCTG -3’ |
| *PPP1R15A* | Forward primer: 5’- GTCCATTTCCTTGCTGTCTG -3’  Reverse primer: 5’- AAGGCGTGCCCATGCTCTGG -3’ |
| *GADD45B* | Forward primer: 5’- CGGCCAAGCTGATGAATGTG -3’  Reverse primer: 5’- GATGTCGATGTCGTTGTCGC -3’ |
| *TRIB3* | Forward primer: 5’- CCATTTGGTCCTGACGGAAAGTCG -3’  Reverse primer: 5’- AGCCTTGAAGTCACAAGCCGTTTC-3’ |
| *MAFF* | Forward primer: 5’- ATCCCCTATCCAGCAAAGCTC -3’  Reverse primer: 5’- TTGAGCCGTGTCACCTCCTC -3’ |
| *ATF3* | Forward primer: 5’- CCT​CTG​CGC​TGG​AAT​CAG​TC -3’  Reverse primer: 5’- TTC​TTT​CTC​GTC​GTC​GCC​TCT​TTT​T -3’ |
| *β-Actin* | Forward primer: 5’- CTTCAACACCCCAGCCATG -3’  Reverse primer: 5’- GTGGTACGACCAGAGGCATAC -3 |
