# Supplementary material for: Single-cell protein activity analysis reveals a novel subpopulation of chondrocytes and the corresponding key master regulator proteins associated with anti-senescence and OA progression
Source: Front Immunol. 2023 Mar 23;14:1077003. doi: 10.3389/fimmu.2023.1077003 (PMC10077735; doi:10.3389/fimmu.2023.1077003)
Supplement: Supplementary file 7 [file Table_2.docx]

**Table S2 Patients’ Information**

| **Patient ID** | **Age** | **Gender** | **Height(cm)** | **Weight(kg)** | **BMI** | **Knee** |
| --- | --- | --- | --- | --- | --- | --- |
| 1 | 71 | female | 160 | 71 | 27.73 | Left |
| 2 | 72 | female | 166 | 75 | 27.22 | Left |
| 3 | 74 | female | 156 | 74 | 30.41 | Right |
| 4 | 68 | male | 168 | 80 | 28.34 | Left |
| 5 | 58 | male | 169 | 92 | 32.21 | Left |
| 6 | 61 | male | 170 | 65 | 22.49 | Right |
| 7 | 61 | male | 168 | 65 | 23.03 | Right |
| 8 | 68 | male | 180 | 100 | 30.86 | Left |
| 9 | 61 | female | 168 | 78 | 27.64 | Right |
| 10 | 66 | female | 150 | 60 | 26.67 | Left |
