# Supplementary material for: Single-cell protein activity analysis reveals a novel subpopulation of chondrocytes and the corresponding key master regulator proteins associated with anti-senescence and OA progression
Source: Front Immunol. 2023 Mar 23;14:1077003. doi: 10.3389/fimmu.2023.1077003 (PMC10077735; doi:10.3389/fimmu.2023.1077003)
Supplement: Supplementary file 8 [file Table_3.docx]

**Table S3 Marker genes of Seurat clusters in GSE169454**

| **Marker genes of Seurat cluster 1** |
| --- |
| *KCNMA1 HMGA1 SOD3 PDLIM4 S100A16 RHOC C2orf40 ANXA2 SEC61B MYL6 S100A6 PMEPA1 BMP2 CRYAB NME1 P3H2 UPP1 S100A13 LRRFIP1 S100A1 PGAM1 PHLDA1 FGF1 TGFB1 ACTG1 GTF3C6 TUBA1B S100A10 FGF2 SYNJ2 CNN3 ISG20 SRM CD55 EIF5A RFLNB EIF4EBP1 SMOX CLMP SPRY2 STK17A CITED4 ACTN4 PDIA6 FDPS SELENOK FGFBP2 GLRX3 BCAT1 SPINT2 CALR FKBP2 SDF2L1 C1QBP PAPSS2 TALDO1 PHB RHOD PLAUR AKIRIN2 EBNA1BP2 HSP90B1 LARP6 CLCF1 AL078639.1 TUBA1C TENT5A PGM3 EMP2 FUS HAO1 PHGDH EIF4A1 TXNRD1 SERP1 SEC11C DCAF13 SOCS2 ODC1 LGALS3 RHOF S100A3 CDA COX7A1 HSPA5 TMX4 UGP2 PDE4B LRP10 NAPG ZFAND5 TAGLN2 CYTL1 GOLIM4 ZMIZ1 SARS DNAJB11 APOD TOMM34 PTPRE MAGED2* |
| **Marker genes of Seurat cluster 2** |
| *LUM CRTAC1 SOD2 CLU FTH1 CHI3L1 CSTB B2M GPX4 NNMT H2AFJ STEAP1 CHI3L2 FTL GLRX SAT1 HTRA1 SERPINE2 CARHSP1 AKR1B1 AKR1C2 IFITM2 OGN MMP3 PNRC1 AKR1C1 ACKR3 H1FX CDO1 CEBPB IFITM1 NUPR1 LAG3 MGP FN1 ABI3BP SOX4 CCPG1 IER3 WTAP CYBA TIMP2 SLC16A7 NBL1 HLA-B SRGN TIMP1 RPS27L ARRDC3 HIST1H1C GALNT15 STEAP2 NPC2 FSTL1 HMOX1 ADAMTS5 C1S PRELP GYPC DEPP1 TNFAIP6 MAN1A1 TWSG1 AGTRAP GBP2 OSMR TMSB4X CD99 C1R MAFB LYRM4 PPDPF CRLF1 PSME1 CTSD NFKBIZ PRDX2 ABRACL MGST3 GCH1 CCDC71L ADAMTS6 ACSL4 TSPO AHNAK HNMT CTSL GRN SLC2A1 GADD45A PPP4R4 IGFBP7 SMOC1 SH3BGRL3 MT1X SCARA3 MT-ND2 RND3 TPI1 ASPN MT1E* |
| **Marker genes of Seurat cluster 3** |
| *SPARC COL2A1 COL9A3 COL9A2 COL11A1 SCRG1 TNFRSF12A COL6A1 PPIB P3H2 COL6A2 CNMD CHAD HAPLN1 COL6A3 ITGB1 CLEC3A CSPG4 ITM2A HMGA1 RPS18 NT5E SQLE ITGA5 SNORC RFLNB MYDGF MSMO1 COL9A1 MANF ANXA5 RRBP1 INSIG1 PPIC PDGFA VASN TAGLN CILP2 SLC5A3 PRSS23 SLC29A1 CD59 PLOD2 DRAP1 SPCS3 PCOLCE2 HNRNPAB SSR3 EMILIN1 TM4SF1 FMOD FLNA PGAM1 RAN MRPS6 HSPA5 PLAUR SET MFGE8 ENPP1 PDLIM4 RCN3 FGFRL1 ACSL3 PDIA3 SDC4 INHBA MCFD2 FGFR1 RBP4 RPL23 PFN1 DNAJC3 P4HB RTN4 LOXL2 CXXC5 IL6ST HM13 PMP22 THBS1 CAVIN1 FRZB TPM1 MT-ND3 TMEM30A OAF GLDN MATN3 COMP P4HA1 PPP1R14B GOLIM4 GSN TKT AEBP1 RCN2 ACTN4 TSPAN2 ANXA2* |
| **Marker genes of Seurat cluster 4** |
| *PRG4 CHI3L2 MMP3 SOD2 CRTAC1 NNMT SEMA3A NAMPT PNRC1 BTG1 DPT LUM GLRX TNFAIP6 SLC25A37 IFITM3 RETREG1 TWISTNB STEAP1 GLUL ERRFI1 SOD1 WTAP C9orf3 MAN1A1 SDCBP SLC7A8 CHST2 ARID5B FNIP2 FOXP1 LTBP1 PKM H1FX ZFP36L1 MAFB C1S HIF1A NDUFV2 PTGES3 FAM110B SEMA3C RUNX1 CREB5 ARAP2 TNFAIP2 TWSG1 PLA2G2A USP12 DDX24 ADAMTS5 MYO10 ANKRD12 OSMR TPI1 MT-CYB MT-CO2 SGK1 TRPS1 SLC2A1 PRRX2 YPEL3 PTGR1 PGD LYRM4 PARP16 HIST1H2AC ZFP36L2 TIMP1 NQO1 ETS2 ACSL4 CSGALNACT1 SOX4 FBXO2 SLC16A10 CYLD THBS4 APP FRMD4B ATP1A1 IFI16 GJA1 AGFG1 PPP1R14C ADGRG2 UGDH DYNLL1 CREBRF LTBP3 NRP2 APOL3 UGCG GFPT2 RB1CC1 SERPING1 PROCR ALDH3A2 KDM7A NDNF ANKRD28* |
| **Marker genes of Seurat cluster 5** |
| *FOS DNAJB1 HSP90AB1 HSPB1 GADD45B JUN CTGF HSPA1B EGR1 HSPA1A HSPH1 HSPA8 HSP90AA1 VIM IER2 DNAJA1 ATF3 UBC DUSP1 BAG3 SRSF7 HSPD1 IER5 LSP1 JUND KLF2 JUNB NR4A1 RSRC2 CALM1 DNAJB4 FOSB EIF4A2 HSPA6 RASD1 GADD45G ZFAS1 TRA2B CLK1 TSPYL2 SNHG12 SOCS3 CRISPLD1 NDUFA4L2 RPL10 DNAJA4 SCX IFRD1 HSPE1 JMJD1C ZFP36 CYR61 CDKN1C UBB MAP1LC3B CDKN1A RHOB MKNK2 SRSF3 ANKRD37 CKB SKIL LDLRAD4 CIRBP SERTAD1 CCNL1 SNHG8 TUBB2B BGN DNAJB6 CKS2 JMJD6 NR4A2 EIF5 TUBB4B RSRP1 TCP1 CHORDC1 CLEC3A BTG1 SNAI1 KLF4 DDIT3 TRA2A GTF2B PLCG2 TSC22D1 THUMPD3-AS1 ID3 CTHRC1 AHSA1 KLF10 LRIF1 ZFAND2A ING1 DUSP2 RPS18 NASP TUBB2A SERPINH1 UBE2S CCDC3* |
| **Marker genes of Seurat cluster 6** |
| *GSPT1 CDC42SE1 SET CAST PPP1CB FAM129B PPP3CA NORAD PTGES3 ZFX NEBL GOLGA4 GOLGB1 PRPF4B FAM133B STK24 HSD17B12 USP12 MINPP1 MEF2A ARHGAP21 CTDSPL CBX1 SEC24D AC092069.1 RNF187 ABHD2 GPC6 CASC4 LARP1 MYH9 MACF1 CCDC6 SPEN GOLGA2 SUMF2 DOCK5 INO80D MPHOSPH10 MXD1 SEC24A SUSD6 GNL3 COBLL1 TNIP1 DIP2C AZI2 KMT2A NDUFAF4 ZBTB1 TMEM41B STX7 ZNF37A CRNKL1 CEP350 LRPPRC METTL14 SIAH2 PSMA3-AS1 MBIP GMPPB DYNLL2 TAOK1 KAT5 PDZD8 ZNF830 WISP3 C3orf52 POR AP001816.1 MBD3 N4BP2L1 AK4 AC139887.2 SEMA7A* |
