# Supplementary material for: Single-cell protein activity analysis reveals a novel subpopulation of chondrocytes and the corresponding key master regulator proteins associated with anti-senescence and OA progression
Source: Front Immunol. 2023 Mar 23;14:1077003. doi: 10.3389/fimmu.2023.1077003 (PMC10077735; doi:10.3389/fimmu.2023.1077003)
Supplement: Supplementary file 9 [file Table_4.docx]

**Table S4 Master regulator proteins of protein activity-based clusters in GSE169454**

| **MRs of protein activity-based cluster 1** |
| --- |
| XRN2 ROCK2 ZFR SQSTM1 RAP1B XRCC5 DYNC1LI1 SPRY2 ZNF385D VCP SPART YWHAB RAB14 CAMK2D SLTM ATP6V1B2 ABCB1 SLC4A7 HNRNPK RAB10 GULP1 BPTF TERF1 AATF MED21 C1D SF1 CDC5L CDV3 RSF1 TIAL1 ATP1B3 STK17A ZNF706 UBE3A MAP4K5 NAA15 SUPT16H NOC2L CERS2 ZFAND6 CSNK2A1 DAB2 CDC42 CEBPZ CCDC47 ELK3 WASF2 MED28 PAK2 DFFA SWAP70 TRIP11 URI1 MGLL FNTA NUFIP1 ZMAT2 TGIF1 COPS2 ESF1 PMP22 NTN4 DEK MEAF6 DAP3 GNG12 RBPJ HNRNPD NELFE SVIL EWSR1 TFG TXNRD1 SP100 PHF5A METAP2 MNAT1 ZPR1 BZW1 BRD7 ADIPOR1 YARS SMC3 LIMA1 SMAD5 FOSL1 LRRFIP1 GLIPR1 ZNRD1 G3BP1 ZNHIT3 RAC1 PLEKHO1 NSMCE3 CDK4 ANKRD11 ZEB1 ABT1 CNKSR2 |
| **MRs of protein activity-based cluster 2** |
| CRYL1 TSPAN4 TMEM25 IFITM1 GADD45G IL11RA RGN ZNF846 TCEA3 CDKN1C GPRC5C TSC22D3 BEX3 NRG2 IFITM3 ETV1 ATF7IP2 CNTFR LY6E PIK3R3 SLC14A1 NDRG2 PLB1 BOC MYBL1 LGALS3 THRA RHOB CD14 ZNF395 SDC2 LSP1 PTH1R POU3F1 FGFR2 RASL11A TMEM8B SOD1 ZNF821 PRDX2 FOS SNAI1 SLC7A8 DDAH2 DDIT3 TLE2 JUND FGFR3 INTS6 HEXIM1 PFDN5 SAP30 CCRL2 TCEAL6 B2M NR4A1 ATP1B2 FCGRT PDE1A TMEM59 UBB MEF2C BARX1 TCEAL2 NLRP1 PRX EPB41L2 CBX4 FXYD1 HOXA5 ERV3-1 MAP2K6 SH3BGRL MYLIP DIRAS3 TNFSF12 HLA-C NFATC1 PLCG2 GNB5 NDUFS4 CKS2 CITED2 HOXC10 TMEM100 DNAJB4 FOSB CALM1 ARL4D RAB17 TCEAL7 ENO2 ZNF385B TIPARP CA2 BNIP3 CAMLG TRIM69 ZNF519 RCAN2 |
| **MRs of protein activity-based cluster 3** |
| PNRC2 CHD2 NDRG1 CRY1 TSPYL2 EMP1 CREBRF HBP1 ETNK1 PDCD4 ARID4B MEF2A MYLIP RYBP CIP2A JMY CCNL1 RRAD ATF3 FOS PEG10 EPC1 PRX DDX17 RFX2 DNAJB4 SCML1 ERN1 HMGB2 MAFA POU3F1 RAB5A WSB1 RASL11A EZR DIRAS3 MYBL1 CHD1 ARID5B SERINC1 ASB3 DNAJB6 MAFF GPBP1 DUSP2 PIK3R3 PPP1R10 BAG3 CALM1 HSPB1 CPE UBL3 FOSB SDC2 KHDRBS3 GADD45B ZFAND2A INTS6 CDKN1A TIPARP JMJD1C EFNB2 SLC16A1 NR4A1 NDRG2 GPRC5A ABCG2 RASD1 DDX5 HBEGF ZNF296 JMJD6 PPP1R15A JOSD1 HLA-C STX3 CYTL1 GADD45G SNAI1 MARCKSL1 PPP2R2A HLA-A H2AFX PRNP CA2 SLC7A8 HLA-E NUDT4 CDKN1C CNTFR TBC1D15 CBX4 PNN ATF7IP2 KATNA1 NFATC1 NKX3-1 TRIB3 UBE2B CEBPG |
| **MRs of protein activity-based cluster 4** |
| PHLDA1 SLC7A5 HDGF MAP4K4 EEF2 MAP4 STK24 ACTN1 GSPT1 PRKDC ILF3 VEGFA ELL2 ATXN10 RASSF8 SND1 LRRC38 SLC38A1 ANKRD11 EPAS1 GDI2 CLMP ZNF503 URI1 LMO4 VASN XRCC5 ZNF385D M6PR SLC4A7 IPO7 PTPN11 CTNNA1 G3BP1 SAFB SMARCD1 AKAP12 KHDRBS1 SRPK1 LPIN2 TRIB1 MALL MED8 PRRX1 GREM1 PTP4A2 LRRFIP1 SRP72 CAVIN1 YARS ATP1B3 CAP1 MYCBP2 XRN2 THRAP3 ZDHHC5 NCOR1 NONO FST USP14 RYK ZEB1 CDC37 DFFA TRIP11 SPEN GLRX2 HNRNPK TFG PPFIBP1 VCP ROCK2 RAB14 RIF1 MAPKAPK2 ZMAT2 SLTM ELF1 ACIN1 BRD7 SSRP1 PRKCA MET STAT3 TCF25 STX6 DDX1 PAFAH1B1 RUVBL1 SLC1A5 RAB18 HTATSF1 PDAP1 MPZL1 PURA MYC PLK2 ZNF638 SNX9 ETS1 |
| **MRs of protein activity-based cluster 5** |
| FAT3 PTK2B ITGAV APP TMED10 PDIA6 CDON CALR PDIA3 CD55 SMOC2 SLC39A14 ZNF628 APLP2 CD46 ITGA5 SLC7A2 CD109 STK38L ANK3 CSPG4 INHBA TIAM2 TNFRSF11B BTG2 LRP1 DNER PDE4B FURIN FRY SDC4 CBLB IL6ST HMGCR DST RAPH1 SLC16A7 FAM129B ITPR3 CLU SIRPA ICAM1 KLRD1 FSTL1 SOD2 ENPP1 VNN3 SDF4 NT5E ACKR3 PSD3 ATP1A1 NRP2 SLC2A1 ITGB1 DSC2 LPP FGF2 FNIP2 FAT1 BHLHE40 TRPV4 GLG1 CLIC4 ELF3 TGM2 IQGAP1 MAGT1 RRBP1 MAP3K6 IL1RN RCAN1 GABARAP BCL2 NFE2L1 MARVELD1 HAS3 RABAC1 ANKH SULF2 WLS ITM2B MSMO1 TBX19 XBP1 ACLY MAP3K8 ZNF385C HIF1A CD63 IFNAR1 SLC20A1 CSF1 ARHGAP42 SOX9 AOC2 CD99 MAML2 PDPN SLC1A1 |
| **MRs of protein activity-based cluster 6** |
| NFIC PPFIA1 FADS1 SPIRE1 GTF2I TRPM7 RAB6A CREB3L2 CDC42BPB GATAD1 TMX1 KDM2A ENAH MAPKAP1 SOCS5 PAFAH1B2 STXBP3 THRAP3 DDX1 PTPN14 ZNF106 NSD1 HTATSF1 FAM120A SND1 DNM1L FLII MED13L PGRMC2 TMF1 ITPR2 DNMT1 CAND1 PRKAR2A UACA MBD2 UPF3A CSDE1 SMC1A SNX13 CXXC5 NRIP1 NCK2 ZFYVE16 CCNL2 GTF2F1 MAP3K2 NFKB1 SETD7 HNRNPUL1 TGOLN2 CDKN2B GOT2 ZBTB38 PDS5A PPFIBP1 ZNF267 SCFD1 DLG1 ANP32A GDI2 KMT5B ARHGEF12 SIPA1L2 CALD1 ABL2 ZFHX3 ARHGAP5 SUPT5H MYO9B EIF4EBP2 NDEL1 NDFIP2 GMCL1 EHD2 ARFGAP3 DDX23 ARID1B CBX5 CUX1 PXK HGS RPS6KA3 WSB2 MACF1 GOPC TRAF3 SMARCA1 TBK1 IGF2R KHSRP STK26 PINK1 COPB1 FZD8 NOTCH2 KIDINS220 MYH9 CIAO1 ITCH |
| **MRs of protein activity-based cluster 7** |
| HDLBP VCL MSN BMP2 ITGB1 PPFIBP1 ATP1A1 CSDE1 WWTR1 CLTC KLF3 MYH9 TMF1 LIF ARFGAP3 IL6ST SOCS2 DST SEL1L RRBP1 PTPN11 HIPK2 MPZL1 BMP6 CBX5 TFRC B4GALT1 CLIC4 DLG1 VSTM4 TGOLN2 COPB1 FLNA CHD9 ARHGAP21 PLEKHA1 ITGA5 HMGCR SLC5A3 RPS6KA3 MACF1 NRIP1 PJA2 GDI2 RAB6A PMEPA1 TGFBR2 ITGB5 TRPS1 PTPRM AFDN DSC2 LPIN2 CLMP HMGCS1 GTF2I TRPM7 SLC20A1 EMP2 PTK2 RAPH1 STK24 AKAP9 CD44 AGO2 WLS KCNK1 SLC30A1 STK38L TMX1 GNB1 STK26 MXD1 IGF2R ZMIZ1 SPTBN1 PRKAR1A ANKH HSPA5 PDIA3 ASAP2 CD46 LPAR1 MAP4 ZFYVE16 ADAM12 APLP2 VEGFA XBP1 MSMO1 SLC1A1 FBXW11 SRPK1 GOT2 MAPK6 PDIA6 PSD3 ACLY ARL1 GLG1 |
| **MRs of protein activity-based cluster 8** |
| PARK7 NEDD8 PRDX1 S100A10 ELOB PFN1 S100A11 FKBP1A TXNDC17 CFL1 POLR2I MDH2 SRI PSMC5 ZNF593 RANBP1 PHB ADRM1 PRMT1 DRAP1 UBE2L3 MRPL12 RHOC EEF1E1 CLTB C1QBP COPRS TXN ARPC2 EIF4EBP1 VDAC1 HMGA1 CCDC124 PSMA4 PSMC3 LAMTOR1 BAG1 ARHGDIA DYNLL1 GTF2A2 GLRX2 RHOA  PDCD6 POLR2K YBX1 ENO1 RAN ACTB PHB2 TNFRSF12A POLR2L PDAP1 PUF60 RAC1 CLIC1 PPP4C FHL2 PA2G4 CLTA RAB34 HSD17B10 CD320 ANXA1 EEF1D RPS3 UBA52 CSNK2B HNRNPDL GTF2H5 YWHAB RPS14 ANXA2 CCDC85B SLIRP PRDX2 RAB5C ATP5F1B ARF5 MGLL CBX3 NACA RPS27A GLIPR1 AP2M1 TAF10 ACTG1 ARF4 GAPDH RPSA CDC37 PRDX3 UBE2I PLP2 ZNHIT1 HINT1 RPL7 MIF APEX1 S100A16 RPL6 |
| **MRs of protein activity-based cluster 9** |
| IFITM3 STC1 B2M SELP IFITM1 MMP2 CDKN1C SLC14A1 GYPC MPP7 IFITM2 RGN SNAPC1 TSPAN4 BMP4 SOD1 RAB17 C2CD4A RRAD RND3 GADD45G RASL11A PMAIP1 BNIP3 BEX3 UBE2B SAP30 PTH1R TCEAL2 RCAN2 MYLIP ATF7IP2 MAFB FOXP1 HBP1 NDRG2 ARL4D INTS6 TSC22D3 LSP1 PLB1 NDRG1 DIRAS3 DNAJB4 POU3F1 CLIC2 ETV1 CRYL1 PTN CKS2 PEG10 TBC1D15 TCEA3 NRG2 RPS27L UBB JUND ZFP36L1 TIPARP ARHGEF33 AQP7 CDC42EP2 MYBL1 RHEB MEF2C TLE2 HMGB2 PDK4 CCRL2 PFDN5 BOC LY6E HLA-A EEF1D ZNF385B KHDRBS3 STX11 SDC2 PNRC2 MARCKSL1 CD14 RPS27A ATF3 DDIT3 SCML1 TNFSF9 CREBRF PIK3R3 HLA-C TNFRSF14 PHLDB2 PLCG2 ADRB2 CITED2 EZR RFX2 BLVRB CHIC2 HOPX FOSB |
| **MRs of protein activity-based cluster 10** |
| ITGB8 NAMPT IFITM2 F5 VCAM1 GPR88 RIN2 PNRC1 ARRDC3 STEAP2 TLR2 TNFAIP3 SELP STEAP1 SLC39A8 SOX4 PMAIP1 ZFP36L1 RASGRP1 ARHGAP28 EREG MARCKS ALDH1A2 MAML2 FCER1G MYO10 SAV1 CEBPD TNFRSF1B FRY CLU FAP OSMR IL6 SIRPA ZP3 NOD2 PTGES NFKBIZ TNFAIP6 NFE2L2 PTGER4 ID2 FNIP2 CARHSP1 HIF1A SLC2A1 TGFBI ZEB2 TOM1L1 VNN1 VNN3 BTG2 CYBA SLC16A7 SLC11A2 CXCL2 ADAMTS1 EPS8 SVIP MAP3K8 IRAK3 DDIT4L LPP DPP4 FLOT2 MAFB ABI1 SOX11 CD38 SGK1 CFLAR IL1RN SOX5 TREM1 NR1D1 NOS2 SULF2 PLD1 SOX9 IFNGR2 NMI SOD2 FSTL1 BIRC3 PDE4B NGF PTK2B FAT3 BASP1 CEMIP PILRA PTPN12 SULF1 KMT2E SLC7A2 MEOX2 TACSTD2 NR1D2 CD274 |
| **MRs of protein activity-based cluster 11** |
| PTPN12 CTNNB1 PRKAR1A ZFAND5 MXD1 EPS8 KLF6 SOX9 AKAP9 ENPP1 ADSS LPP FNIP2 FAT1 PSD3 ABI1 CD109 PDE4B ARHGAP42 PJA2 LRP1 ARRDC3 CD55 SDF4 ANKH BCL2 TIAM2 ID2 NFKBIZ GABARAP SDC4 CSF1 SELENOK ADAMTS1 DST IQGAP1 ANK3 SERTAD2 SMOC2 TGM2 NR1D1 FBXW11  FSTL1 NFE2L1 APLP2 DAP SLC16A7 APP SLC7A2 ITGAV CLU IRF4 NRP2 BTG2 DNER SGK1 MAP3K8 CLIC4 ITPR3 OSMR SLC5A3 MARVELD1 VNN1 AOC2 NFE2L2 DDIT4L LPAR1 CEBPD BHLHE40 CHD9 SLC39A14 RCAN1 TRPV4 HIF1A TUSC3 ICAM1 TCF7L2 FAP SLC20A1 BIRC3 MDM2 IFNAR1 TNFRSF11B CD46 IL13RA1 OPTN SMOC1 KCNK1 ASPH NOS2 NT5E SULF1 GAS1 IL6ST FGF2 IFNGR2 ATP1B1 SLC2A1 MAPK6 SOD2 |
| **MRs of protein activity-based cluster 12** |
| AGTRAP TBC1D22B LCORL IL6 MLLT3 SIRT5 BATF IL11 ASCC2 PSTPIP1 RNF20 TAF1B GMNN TSC2 ZKSCAN8 IRAK3 HMG20A KDM4B ZNF398 ARID2 MTMR1 ZNF3 IRF2BP1 TBX18 TSHZ1 ARHGEF9 ZNF431 C2CD3 PDE1C KLHL12 ZNF334 PRDX4 NFATC3 CNOT6 ZNF621 ATR ZBTB33 SUPT3H NEDD1 ZNF444 EIF2AK3 SNAPC3 ZFYVE1 TFB1M CD99L2 ZNF512 MTERF1 ZDHHC17 INPP5A JAZF1 LDLRAD3 ZNF460 POU2F1 EFS PBX1 ZNF605 SCRIB CC2D1A FMN1 NACC1 PPRC1 CPTP ZZEF1 ZNF346 RHBDL2 MED26 ZFAND2B KCTD7 ZMYND8 HAND2 TDRD3 GCC1 YEATS4 TCF4 ARL5A SLC13A3 PILRA LIN54 ZNF335 CIC KLRD1 TNP1 ZNF354C NAB2 FRAT2 ZNF362 ARHGEF6 YEATS2 ZNF569 TAF6L STAT5B CSNK1G1 NELFA TAF6 INPPL1 RAB29 ZBTB2 NDP ZNF470 NIF3L1 |
| **MRs of protein activity-based cluster 13** |
| TREM1 IFITM2 F5 PMAIP1 GPR88 CARHSP1 STEAP1 ITGB8 SELP ATRAID RIN2 UCHL1 SVIP PRRX2 TMEM219 TLR2 ITM2B AGTRAP FLOT2 SOX4 ANGPTL6 HHIP ZP3 TOM1L1 HAS1 NAMPT MARCKS PTGES CYBA TGFBI SLC39A8 NDP FCER1G NGF CD81 VCAM1 NR1D1 MGST3 TACSTD2 ARHGAP28 RASGRP1 THY1 CLU TMEM59 STEAP2 LGALS3 VNN1 CD38 SOX11 VAMP8 TSPO TNFAIP3 BDKRB1 CEBPD EREG CYBRD1 PNRC1 PILRA MSRB2 VNN3 ALDH1A2 FRY BATF NMI FXYD6 GRN GSC PLD1 TNFRSF1B ARRDC3 SIVA1 NTF3 IL6 DUSP1 IL1RN RGS2 TPD52L1 FAP HOXC6 BASP1 PRKAR2B CA12 ZDHHC12 TNFAIP6 ZEB2 PPIC SH3KBP1 LAG3 ECM1 ITGAE DDIT4L TMEM50B RGS16 SYT11 CDC42EP5 IGFBP2 PTGER4 ZFP36L1 ANG NOD2 |
| **MRs of protein activity-based cluster 14** |
| PLAUR IL11 CCL2 EMP2 RGS3 EDF1 ITGB1BP1 FGF1 GSTP1 IGFBP3 EMP3 ARL1 SNF8 HSBP1 ARHGAP44INSIG1 PMEPA1 CXCL14 S100A16 MSMO1 AP2S1 SRA1 S100A1 TNP1 CD63 EIF4EBP1 DIO3 SLC9A3R2 LOXL2 MAP2K2 S100A6 VPS28 CA9 CSDC2 KCNMA1 SLC2A6 HSPA5 RHOD WNT7B HAND2 PRDX4 CITED4 PDIA3 RHOC INHBA EPB41L3 PDIA6 ANGPTL4 STRA6 CALR PSTPIP1 VPS25 CD320 HMGCS1 HMGCR KCNN4 FLNA AMIGO2 GAL EID1 LRRC8C PTK7 CDH19 CAV1 ITGA5 IGFBP6 DOC2B KLRD1 TBX19 ARL3 SLC29A1 SELENOK DNAAF1 HR DUSP4 GRIN2A AOC3 NRGN ATP1B1 ERBB2 CIB1 SIRT6 ANOS1 GOLT1B MPZL2 LRRC8D DYSF KCNG1 TP53I13 AQP1 CAVIN1 ACAP3 CFB PTPN6 DCHS1 SELENOS KRT17 STK38L CD24 DHCR24 |
| **MRs of protein activity-based cluster 15** |
| SLC9A3R2 EMP3 CIB1 HSBP1 EDF1 SRA1 CLIC3 PLAUR AP2S1 GSTP1 ITGB1BP1 CNIH4 CD151 OSTF1 ARL1 ARL3 MAP2K2 CCND1 RGS10 S100A16 SNF8 SELENOS CAV2 VPS28 VPS25 CD320 SCAND1 PSMA4 IGFBP6 MGST3 NRGN STUB1 SLC39A3 S100A6 GOLT1B YIPF3 ZNHIT1 CHURC1 ENG AP3S1 HMGN1 HM13 S100A1 COPRS HINT1 CD63 CDC42EP5 KCNN4 TP53I13 RGS3 DCHS1 ATP1B1 FIBP LRPAP1 RHOD CALM3 WSB2 KCNMA1 STRA6 FOXC2 CDA FGF1 NEDD8 GPC1 SLC29A1 TMX1 ID1 CXXC5 TAF10 TFPI DERL1 SERPINE1 PIN1 TCEAL3 SIVA1 CXCL14 RHOF PSENEN CDC42SE1 GAL TUSC3 DERL2 RHOC WNT7B INSIG1 GNAI2 CCL2 DIO3 TSPO KCNK1 MYADM GNG11 ARF5 DUSP4 PRDX4 RAB40C LOXL2 TCEAL8 MRGPRF EID1 |
| **MRs of protein activity-based cluster 16** |
| FGF2 INHBA PDIA3 PDIA6 CALR CD44 MDM2 DSC2 IRAK3 NFKBIZ HMGCR TIAM2 NOS2 BCL2 ICAM1 ID2 IL11 ATP1A1 HAS3 ALDH1A2 BTG2 CD46 TNFRSF11B CLIC4 CSF1 MAML2 IL6 SLC39A14 PDPN SLC7A2 STK38L KLRD1 IGFBP3 SOD2 GJB2 RCAN1 ZEB2 PSD3 SIRPA SEMA6D DNER ITPR3 CD82 SLC16A7 LPP NRP2 APP PTK2B SOX5 MTMR1 LIF ZNF628 SDC4 ITGA5 DST CXCL2 PDE1C TNFAIP6 CD55 SULF2 MYO10 MEOX2 EREG VCAM1 HSPA5 MAP3K8 VNN3 CXCL3 ITGAV NFKBIA ANK3 ARHGAP28 HIF1A FNIP2 SOX9 ARHGAP42 MAGT1 TRPS1 STEAP2 RIN2 NOD2 ACKR3 PTPRM FSTL1 FAT1 IL6ST SLC1A1 PDE4B CD109 FAT3 TGM2 SMOC2 FAP IRF4 MSMO1 CXCL14 NFIL3 CDH19 FRY TCF4 |

MRs: Master regulator proteins.
