# Supplementary material for: Single-cell protein activity analysis reveals a novel subpopulation of chondrocytes and the corresponding key master regulator proteins associated with anti-senescence and OA progression
Source: Front Immunol. 2023 Mar 23;14:1077003. doi: 10.3389/fimmu.2023.1077003 (PMC10077735; doi:10.3389/fimmu.2023.1077003)
Supplement: Supplementary file 10 [file Table_5.docx]

**Table S5 Leading edge proteins of protein activity-based cluster 3 and 13 in GSE169454**

| **Leading edge proteins of cluster 3** |
| --- |
| JMJD6 CREBRF SLC16A1 NFATC1 PIK3R3 HBEGF PPP2R2A ZFAND2A CHD2 TBC1D15 NDRG2 PDCD4 WSB1 GADD45G RASL11A CDKN1C SLC7A8 ERN1 SCML1 ARID5B RYBP DIRAS3 NDRG1 BAG3 TRIB3 JMJD1C MYLIP CEBPG PEG10 JMY TSPYL2 CYTL1 HMGB2 RASD1 CCNL1 TIPARP NR4A1 GPRC5A FOS PPP1R15A GADD45B CDKN1A FOSB MAFF ATF3 DUSP2 |
| **Leading edge proteins of cluster 13** |
| TREM1 THY1 TNFAIP6 TGFBI SOX11 NGF BASP1 CA12 FAP ITGB8 ECM1 PPIC SYT11 MARCKS PTGES VCAM1 SH3KBP1 TACSTD2 STEAP1 GPR88 ALDH1A2 ARHGAP28 F5 SELP PLD1 RGS2 FCER1G TOM1L1 CYBA CD38 HOXC6 PTGER4 MGST3 NDP GRN GSC FXYD6 |
