# Supplementary material for: Single-cell protein activity analysis reveals a novel subpopulation of chondrocytes and the corresponding key master regulator proteins associated with anti-senescence and OA progression
Source: Front Immunol. 2023 Mar 23;14:1077003. doi: 10.3389/fimmu.2023.1077003 (PMC10077735; doi:10.3389/fimmu.2023.1077003)
Supplement: Supplementary file 11 [file Table_6.docx]

**Table S6 Marker genes of Seurat clusters in GSE152805**

| **Marker genes of Seurat cluster 1** |
| --- |
| *SMOC2 COL9A3 COL9A2 COL2A1 HAPLN1 FMOD MYADM COL11A2 ITM2A SDC2 SPARC MATN3 FOS CILP2 NDUFA4L2 COL11A1 GAPDH JUND FOSB KLF4 JUN LMNA ENO1 EMP1 RGCC LSP1 SCRG1 TUBB2B NRN1 COMP CLEC3A TUBA1A ANXA1 CTHRC1 DNAJB1 RARG FIBIN C16orf80 HSPA8 MAGED1 RGS3 RARRES2 QSOX1 FAM162A MDFI NFATC2 DNAJA1 MLF1 IFITM10 GPC6 P4HA2 FGFRL1 KLF2 HCFC1R1 PYCR1 EPS8L2 COL9A1 MIR24-2 BRD2 CLEC11A SERTAD4-AS1 UGP2 RPL22L1 UGDH SRSF7 PTP4A1 LEPREL4 PLOD1 PPP1R10 WWP2 PLA2G2A COL3A1 SERTAD1 NFATC1 ERF ZCCHC17 ATP1B1 NR1D1 HSPA1B GOLIM4 LDLR PALMD WISP3 RHOB BNIP3 INSIG2 GOLGB1 ATF3 HSPD1 CSRP1 CREB5 WSB1 NOL3 HMGCS1 EGR1 MCL1 TSPYL1 RBBP6 ITIH6 CALR TPM3* |
| **Marker genes of Seurat cluster 2** |
| *CHI3L1 NNMT CLU IFITM3 MALAT1 IFITM2 CHI3L2 BTG1 OSMR ITGA10 SERPING1 AEBP1 HLA-E SLC40A1 GBP2 C1R C1S SCARA3 MT-CYB CNN3 TPT1 SLC25A37 MAFB NDRG1 EFEMP1 TSPAN4 DDR2 SLC39A14 TCF4 COL12A1 YBX3 APLP2 PPP3CA ARHGAP29 PIK3R1 ZNF385D CFH AMD1 ENO1 TMEM173 TSC22D1 HIF1A PMP22 PLSCR1 LBH MT2A NPC2 ERRFI1 SNHG8 BRD2 EPB41L2 SERTAD1 LAG3 ARID5B ADIRF TGFBR2 CAST ANKRD12 HEXIM1 RUNX1 C1orf63 CCDC85B TAF1D GLRX SOCS3 SLC25A5 PPAP2B CTGF CSRNP1 VIM MT1M C5orf15 UBC SERPINB1 GALNT15 ISM1 WSB1 SH3BP5 ELL2 PLA2G2A STEAP4 MT1A CLK1 ASS1 SPOCK1 FOXP1 MT1E ARID5A STOM VCAM1 TXNIP TUBB4B LTBP2 TACC1 EPS8 H2AFX HBP1 ABCA5 RHOBTB3 CDO1 PDLIM4* |
| **Marker genes of Seurat cluster 3** |
| *CRTAC1 OGN S100A4 TNC HTRA1 SMOC1 DPT COL6A2 CDON ABI3BP COL6A1 COL6A3 TNXB TGFBI VCAM1 LUM CLU IGFBP5 COL15A1 ASPN ADAMTS6 F5 LTBP3 CRIP1 COL3A1 GAS1 CD81 KAL1 THBS3 PCSK1N PRG4 PFKP COL1A2 ECM1 CD109 COL5A1 PART1 FN1 MRC2 TMSB4X CHI3L2 AKR1C2 NTN1 SEMA3A P4HA3 ANKH CAPS ISLR COL5A2 TAGLN2 TCF4 GLI3 SIPA1L2 S100A6 GPR64 CRIP2 GRN THY1 PRKCDBP PTGES NDNF MT-ND3 A2M DIO2 C10orf54 ITGB5 NBL1 PDLIM3 MT-ATP6 S100A11 COLGALT2 LAMB2 THBS4 SBSPON ZEB2 VEGFA TIMP2 PTPRD MT-ND2 APLP2 C10orf105 LTBP4 ACKR3 TRPS1 TPPP3 SLC39A14 CREB5 HSPG2 C9orf3 SULF2 KIF13B MT-CYB ITGB1 NT5E ANGPTL2 MT-CO3 RPS12 FAP SOX5 FSTL1* |
| **Marker genes of Seurat cluster 4** |
| *ACTB ACTG1 PCOLCE2 CYR61 STK38L ACAN PPP1R3C TF DCN ITM2C CLEC3A SERPINA1 PRELP NR1D1 RASD1 NR4A1 CILP MT1X BHLHE40 CYTL1 STC2 TPM4 GLIPR1 LDLRAD4 CD9 CSRP1 MGP IGFBP7 MT1E PLA2G2A SCIN NR4A2 TRPV4 FTL CNN2 RBP4 MYL12A IDI1 ADRB2 GDF10 INSIG1 MATN3 DDIT3 CFH MSMO1 NDRG2 MCL1 PID1 JUNB ZBTB16 BTG2 SERTAD4 TSPYL2 NRBF2 AP3S1 TUBB2B LMO4 NUPR1 STK17A ENHO PAPSS2 SLC20A1 PHLDA1 ALDOC SLC39A8 PRRX1 WWP2 KLF6 SERTAD4-AS1 DUSP1 PRPSAP1 ILF3-AS1 ITGA5 ID2 MIR497HG ADSSL1 SRGN ORMDL3 SNAI2 OMD TSPAN2 FDPS P4HA2 TAGLN MEG3 TIMP4 INHBA ERRFI1 JMJD6 FHL2 CTA-29F11.1 HES1 CCNL1 RP11-983P16.4 GALE FAM133B DLG1 AVPI1 METTL7A WIF1 ATF3* |
| **Marker genes of Seurat cluster 5** |
| *C2orf82 FGFBP2 MIA PDPN SSR4 S100A1 CHAD C2orf40 TSPO TSPAN13 IBSP PLAC9 SPINT2 COL10A1 NUCB2 SCRG1 CD99 SLC29A1 TF FN1 PRDX4 LECT1 CRISPLD1 SERPINA1 MFGE8 RAMP1 S100B IER2 TIMP3 JUNB TNFRSF11B IGFBP6 CST3 CD59 APOD FGFR1 CPE FXYD6 ACTB EMP3 HEXB PCOLCE2 TSPAN6 CYTL1 MEF2C SERPINI1 CRYAB MFI2 IVNS1ABP LDLRAD4 CYSTM1 PHOSPHO1 FXYD1 S100A13 AP3S1 SOD3 MDFI RBP4 GLIPR1 AQPEP ACTG1 NTAN1 NTRK2 MRPS6 CALM1 ACAN ADRB2 TTLL7 ITM2C RHOC HSP90B1 NPC2 SPP1 CD164 PLP2 AC005152.3 WISP3 BHLHE41 CAPG CAV1 MYL12A RHOB CCDC88A H1F0 CKB CAPN2 SERPINA5 CLEC3A SLC44A2 KDELR3 CST6 SCIN SEC11C FOXA3 CCDC80 RHOD EGR3 SRPX2 DBI NEBL SMAD7* |
| **Marker genes of Seurat cluster 6** |
| *NFKBIA SOD2 ICAM1 IER3 IRF1 NFKBIZ RGS16 ZFP36 PNRC1 PPP1R15A FTH1 RND1 MAP3K8 ZC3H12A REL SQSTM1 BIRC3 GADD45B MMP3 MAP2K3 SLC25A37 SDC4 WTAP TIFA KDM6B TNFAIP8 BIRC2 SLC39A14 NFE2L2 BAZ1A PIM3 MTHFD2 TNFAIP2 CCL2 GEM MAFF GPX1 RCAN1 ID2 TPBG RELB CEBPD NFKB2 CD44 TPT1 BTG2 NAMPT KLF10 NINJ1 S100A11 GLUL GBP2 NFKBIE SLC7A2 CCNL1 PIM1 TM4SF1 GCH1 RUNX1 JUNB C1orf63 HMGA1 RP11-317P15.4 G0S2 PLK3 SAT1 PHLDA1 DDX21 CSRNP1 CSF1 CYCS CD83 ACSL3 CNN3 NFKB1 BTG1 LIF GPX3 DNAJB9 SLC1A5 TGIF1 TOP1 ZFAND5 RGS3 ASS1 YME1L1 SERPINB1 C6orf62 CALD1 FOXO3 RAB27A CHD1 TAF9 C11orf96 SOX9 CDC42SE1 RBBP6 NFKBID CDC37L1 H2AFJ* |
