# Supplementary material for: Single-cell protein activity analysis reveals a novel subpopulation of chondrocytes and the corresponding key master regulator proteins associated with anti-senescence and OA progression
Source: Front Immunol. 2023 Mar 23;14:1077003. doi: 10.3389/fimmu.2023.1077003 (PMC10077735; doi:10.3389/fimmu.2023.1077003)
Supplement: Supplementary file 12 [file Table_7.docx]

**Table S7 Master regulator proteins of protein activity-based clusters in GSE152805**

| **MRs of protein activity-based cluster 1** |
| --- |
| NRN1 GREM1 NPM1 PPP1R1B RPS27A BOC PFDN5 NACA RPL6 EEF2 RPS6 RPS3 RPS14 GPC6 IGF2 UBA52 RPL7 EEF1D SNAI2 CDK18 PTGER3 DUSP2 BNIP3 DDR2 ASPH CAMLG RPSA FGFR2 DDX3X TRPV4 THSD7A SOX9 MPP6 DUSP1 BTF3 DKK1 LSP1 ABCC6 MMP16 TSC22D1 RHOB RARG HLA-B ITSN1 FGFRL1 PDCD4 SLC26A2 SDC2 MDFI KLF6 GPC5 HOXC10 TPD52L1 HMGCS1 FOXC1 CNTFR VASN SLC22A11 RCAN2 CYBRD1 LMO4 ITM2C DLX5 GPRC5C BLM NTRK2 TCEAL5 NEU1 HLA-C EPB41L3 FXYD1 CPE CRIM1 SLC39A8 ZCCHC17 PRKCZ KLF2 NFATC1 BIRC2 DDX17 NFIA FCGRT ID1 JUN TCEAL8 PDE3B RAB28 SF1 ITPRIP NFATC2 NONO JUND ZRANB2 ID3 PER2 FOXO3 ZKSCAN1 SCG5 LITAF CLCNKB |
| **MRs of protein activity-based cluster 2** |
| ITM2C CYTL1 TMEM59 S100A1 GLIPR1 TSPAN6 PDPN STK38L CD99 ATRAID INSIG1 LRPAP1 FXYD1 ADRB2 PDIA6 MDFI TMED10 FCGRT STK17A SLC29A1 PEBP1 SLC22A17 PRDX4 DAP GSN CRYAB MAP2K6 SDC2 CD320 PKM LMCD1 NDRG2 CD9 PLEKHB1 SPOCK3 PDIA3 TRPV4 RAMP1 SLC20A1 SDF4 ITM2B FXYD6 FGF1 ARF4 MSMO1 IGFBP6 CD59 KCNMA1 EGR2 FXYD2 BHLHE40 AP3S1 MEF2C STRAP ZNF816 SLC44A2 PIM1 DKK1 SLC35B2 HSPB1 TCEAL2 IFI6 DUSP2 HLA-A ACTG1 VASN SDC4 EID1 BHLHE41 EGR3 TMED4 BSG RHOD LYVE1 RASD1 FGFR1 RHOB TUSC3 HERPUD1 RAB1A FOXA3 CPE ACTB SLC7A5 NR1D1 VOPP1 LGALS3 ARL3 RELN VAMP2 ANXA7 BLM HES1 TSPO NR4A3 LITAF RHOC AKAP12 HSPA5 SLC22A11 |
| **MRs of protein activity-based cluster 3** |
| HERPUD1 EMP2 TERF2IP UBC MAP2K6 CD14 HSP90AA1 XBP1 SQSTM1 EIF5 BAG3 PHLDA1 TIPARP HSPA8 DNAJB6 SLC3A2 RBM39 BHLHE40 DDX5 DDIT3 HSP90AB1 LYVE1 CCNL1 SRSF2 NDRG2 HNRNPK PPP1R10 SNAPC1 ARID5A TAF7 LAG3 HNRNPDL STK17A HMGB2 ICAM1 ZNF816 CNBP TSPYL2 DDIT4 GEM SFPQ VAMP2 TCEAL2 HES1 SERTAD1 HMGN2 H2AFX MAP3K8 MAFF HEXIM1 SAP18 ETS2 UBE2D3 RGS3 PIM1 KLF10 SLC1A5 CYCS ELL2 CSRNP1 ZNF385D PPP1R15A LMCD1 GADD45B PNRC1 C4BPA ATF3 EPHB6 PPP1R1C CDKN1A JMJD6 EPC1 BTG2 TSC22D1 ZFAND5 TCEA3 HSPD1 PRNP PTGES3 JUNB WSB1 ADM IRF1 UBB RGS16 SNAI1 CCNH PNN ATF4 SLC14A1 CREG1 BTG1 PPP2CA VGLL4 GSN DACT1 ANXA7 ZBTB16 YBX1 ZNF622 |
| **MRs of protein activity-based cluster 4** |
| BTG1 OSMR LBH DIO3 MAFB HLA-E HMOX1 SQSTM1 HSPD1 LAG3 TXNIP ITGA10 DDX5 ICAM1 PTGES3 EIF5 STEAP4 PLSCR1 PNRC1 EIF2A HEXIM1 SRSF2 SOD2 TMEM173 DNAJA1 CCNL1 SVIP IRF1 SERTAD1 SLC3A2 UBE2D3 RGS16 RBM39 PIK3R1 MAP3K8 HMGB2 HMGN2 HNRNPDL NFKBIZ SNAPC1 HSP90AB1 ZNF347 ARID5B C4BPA HSPA8 H2AFX BAG3 SOD1 NAMPT EMP2 CEBPD CAMLG IFITM3 IFITM2 CCDC59 THBD CNBP ZNF385D SLC40A1 BIRC3 PDK4 NFKBIA HNRNPK ATF4 HSP90AA1 PNN GADD45B FLOT1 BST2 XBP1 TGIF1 ELL2 UBC TIPARP IGBP1 NPM1 ARID4B SF1 ZNF441 PPP1R10 ARID5A CD14 PPP1R15A CHD1 RGS11 ITGA9 DNAJB6 TERF2IP JMJD6 SFPQ SOCS3 SAFB2 DDIT3 SAP18 ERG ZFAND1 ZNF823 NDRG1 HBP1 ANGPTL4 |
| **MRs of protein activity-based cluster 5** |
| TNXB ATP6AP2 CD151 CRABP2 CDA MGST3 CLU ANXA2 CIB1 ECM1 HAS1 AP2S1 TREM1 LYVE1 DPP4 WNT5B TXN LRRC8C BZW1 CD55 HLA-DRB1 SPARCL1 TWIST1 S100A11 PRDX1 DIO2 KCNN4 ITGB5 AHR PLP2 S100A6 TSHZ2 TBC1D2 LIMK1 BMP4 ZEB2 MRGPRF PDLIM1 PLXDC1 AHNAK SLC6A6 LIMS2 CADM1 THY1 NT5E BASP1 PLAUR DYSF LGALS3 NDUFA13 MMP2 CCND1 ANXA4 FZD10 ADTRP CLIC1 ANKH MAP1B CD109 SPRY1 ITPR3 F3 PMP22 CDH13 RAB31 PTGES HMGB1 DPYSL2 POLR2L RTN4 DYNLL1 ANO1 NOTCH3 CAPS CAPNS1 SLC38A5 PROCR CYBA IQGAP1 TNFAIP6 FKBP1A YWHAB GAP43 PKM AP2M1 FNIP2 PFN1 AQP1 NTF3 DUSP4 FHL1 TNFRSF12A CTNNA1 SMOC1 HM13 CAPN2 BID HSPG2 FSTL3 CLTB |
| **MRs of protein activity-based cluster 6** |
| VCAM1 SLC39A14 IFITM3 PMAIP1 F5 IFITM2 NTF3 SOX11 ANK3 ANXA4 TCF4 TGFBI ZEB2 TXN ECM1 DIO2 CLIC1 PMP22 AHNAK FAP BMP4 TWIST1 AQP1 THY1 ADTRP DLX4 CCND1 VIM PDLIM1 NT5E SPARCL1 SLC16A10 GFRA2 CDH13 ADCY7 GLI3 SLC6A6 PLAUR PTGES CD55 RPS27L S100A11 SPRY1 GJA1 MARCKS PROCR SLC38A5 MMP2 WNT5B IGF1 SYT11 TSHZ2 TNFAIP6 NOTCH3 GNG11 IGFBP3 F3 MGST3 PLP2 ANTXR1 HMGB1 DIRAS1 TMEM100 NDRG1 KCNJ15 PREX2 RAB13 FSTL1 BID PLXDC1 FNIP2 DGKI SFRP1 EPB41L2 CLU CAPS STEAP4 ARID5B CNIH1 KCNN4 SOX4 SMOC1 GRB10 NRP2 SFMBT2 FZD10 CD68 PHLDB2 POLR2L BASP1 AHR SULF1 TRPS1 RTN4 LRRC8C IQGAP1 HLA-DQB1 ITGB5 CD109 DPYSL2 |
| **MRs of protein activity-based cluster 7** |
| DKK1 KCNMA1 FXYD1 CPE RAMP1 FCGRT SPOCK3 AP3S1 PEBP1 PDPN BHLHE41 PLEKHB1 GSN DAP MEF2C DUSP1 BLM SDC2 HLA-A CD99 CYTL1 NFIA TRPV4 MDFI SLC44A2 SLC22A17 DUSP2 CD9 TPD52L1 CD320 FGFR1 S100A1 FOXA3 EGR3 PTGER3 NDRG2 ADRB2 TMED10 TMEM59 INSIG1 HLA-C CYBRD1 GPC6 RHOB ZCCHC17 SOX9 SLC20A1 NR4A1 TMED4 NR4A3 SNAI2 TSPAN6 VASN SLC14A1 B2M CD59 ATRAID CALM1 GLIPR1 ITM2C FXYD2 FKBP8 EGR2 SH3BGRL RELN RCAN2 IFI6 LRPAP1 PDCD4 ACTB HMGCS1 ACTG1 THSD7A RHOC SDF4 BOC MPP6 CRYAB RASD1 IGFBP6 PDIA6 CCDC88A PRDX4 PPP1R1B NR1D1 RARG EGR1 ZFAND5 LMO4 GPRC5C ZNF385B MSMO1 CRIM1 SLC35B2 PACSIN3 SLC22A11 BSG VGLL4 TCEAL3 FOS |
| **MRs of protein activity-based cluster 8** |
| DNAJA1 SERTAD1 PPP1R15A HLA-E DDX5 DIO3 CCNL1 SRSF2 HEXIM1 SOCS3 MYADM PIK3R1 PNN OSMR KLF4 ATF4 BTG1 NAMPT HNRNPDL ITGA10 NPM1 HMGB2 TXNIP RBM39 HMGN2 EIF5 SF1 ID1 DDX3X CSRNP1 PTGES3 HSP90AB1 HMOX1 KLF2 PNRC1 PPP1R10 SLC3A2 BST2 ICAM1 EMP1 TSC22D2 ARID4B SQSTM1 TMEM173 UBE2D3 JUND ELL2 EIF2A HSPA8 CDKN1A CHD1 ARID5B STAT3 PLSCR1 LBH FOSB YBX3 WSB1 MAFB JUN ID3 IRF1 CYCS SOD2 ARID5A STEAP4 FGF7 SAP18 FOS VIM ERF C4BPA NEU1 SNAPC1 JMJD1C CNBP DDIT3 GPRC5A CCDC59 TOB1 ZFP36L1 JUNB HNRNPK BTF3 SAFB2 LAG3 ATF3 METAP2 HSPD1 RGS16 XBP1 NFATC1 CAMLG GEM IGBP1 H2AFX BAG3 FLOT1 EGR1 GADD45B |
| **MRs of protein activity-based cluster 9** |
| FXYD6 SLC29A1 TNFRSF11B PMEPA1 AK1 TNFSF11 PRDX4 TSPO RABAC1 STK38L TMED10 DNER ITM2B EMP3 S100A6 CD59 ARL1 FGF2 CXCL14 GLIPR1 S100A1 SGK1 CD63 TUSC3 TSPAN6 CDA IL11 ANKH GSTP1 PRDX1 PKM PARK7 ATRAID LGALS3 BSG CLIC3 SERPINE1 CALR SLC35B2 NGF ATP1B3 HM13 PDIA6 YIPF3 CD320 TMEM59 CD151 FXYD2 EPS8L2 PDIA3 PRDX2 ARL3 TREM1 IGFBP6 DYSF LRPAP1 HDLBP EID1 ARPC2 CRYAB MAGED1 HSBP1 INHBA S100A10 KCNN4 TCEAL3 SLIRP CYBA AOC2 PLAT SLC7A2 CAV1 RHOD HLA-DRB1 GAPDH SLC6A12 CAPN2 TNXB TMEM219 CD99 SMOC2 CAPS CD68 LOXL2 ANXA2 ENPP1 ATP6AP2 ANXA5 RHOC DYNLL1 CD8B DPP4 KCNS3 CAPNS1 CNIH4 TNFRSF12A LIMS2 PLP2 AP3S1 MDH2 |
| **MRs of protein activity-based cluster 10** |
| GAS1 GAPDH CLCNKA NGF ATP1B1 GFRA2 RPSA RCAN1 TNFRSF11B SLC7A2 TNNI2 SMOC1 DST DNER IGFBP3 DOK1 RRBP1 SMOC2 PTGES SERPINE1 UBA52 FSTL1 CA9 CD68 SLC16A10 RPL7 GRN INHBA TRPS1 EEF1D CXCL14 NT5E PPIC RPS14 CNIH1 PLAT TNFAIP6 IL11 PMP22 TGFBI PROCR CLIC3 TNFRSF12A SOX11 RPS3 SULF2 THY1 ADAM12 LOXL2 FNIP2 CDON FAP ANKH AQP3 RPS6 SPRY1 CAPS RAB13 RPL6 NACA GDF5 EPS8L2 ANK3 DIO2 GNG11 CCND1 CLCNKB PFDN5 F5 ZCRB1 IFITM10 AQP1 AP2M1 TRIM29 KCNS3 S100A10 RPS27A FGF18 FHL1 AOC2 SLC38A5 HMGN1 GLI3 CAV1 PKIG ADTRP ADAMTS1 EVI2A DGKI LRRFIP2 KCNN4 PLAUR ENOX1 SLC6A6 ENY2 EDF1 RPS27L SDC1 SLC2A12 CD55 |
| **MRs of protein activity-based cluster 11** |
| CRABP2 MMP2 AHR BASP1 CALD1 PLXDC1 MARCKS HAS1 SPARCL1 TXN TSHZ2 RAB31 AHNAK TNXB CA12 CDH13 ANO1 LIMS2 GPR153 ECM1 CERCAM CADM1 BID FKBP1A MAP1B LGALS1 MGST3 RHOA PFN1 DPP4 DYNLL1 SULF1 CAPN2 CIB1 DPYSL2 AQP1 SAMHD1 TWIST1 CLIC1 HMGB1 IGF1 S100A11 NOTCH3 PDLIM1 ZEB2 ANXA2 PLEC MEOX1 F3 GAP43 CCND1 ITGB5 MRGPRF FLNA AP2S1 S100A16 PSD3 CD109 ARPC2 GNB1 PEAK1 EZR RPS27L FZD10 FGF10 ITGB1 POLR2L YWHAB HSPG2 ANXA4 ADCY7 WNT5B TREM1 PPP1CA TSPAN15 PTGER4 ARL2 SLC38A5 PIN1 MAP4 DUSP4 NDUFA13 CLEC2B SLC6A6 CFL1 DDAH2 ANTXR1 KCNN4 CAPNS1 HSBP1 ENTPD1 IFI27 AXL CCDC124 GJA1 DYSF GPX1 EHD2 DIRAS1 RTN4 |

MRs: Master regulator proteins.
