# Supplementary material for: Single-cell protein activity analysis reveals a novel subpopulation of chondrocytes and the corresponding key master regulator proteins associated with anti-senescence and OA progression
Source: Front Immunol. 2023 Mar 23;14:1077003. doi: 10.3389/fimmu.2023.1077003 (PMC10077735; doi:10.3389/fimmu.2023.1077003)
Supplement: Supplementary file 13 [file Table_8.docx]

**Table S8 Leading edge proteins of protein activity-based cluster 3 in GSE152805**

| **Leading edge proteins of cluster 3** |
| --- |
| NDRG2 WSB1 CCNH BTG1 VGLL4 ARID5A ZBTB16 BAG3 ICAM1 SNAPC1 SERTAD1 ELL2 RGS16 JMJD6 SLC3A2 HERPUD1 ZFAND5 UBC TSPYL2 PNRC1 PIM1 JUNB HMGB2 CCNL1 TIPARP IRF1 ETS2 PPP1R15A KLF10 BTG2 HES1 BHLHE40 DDIT3 CSRNP1 GADD45B TRIB3 MAFF ATF3 DDIT4 ADM |
